# Supplementary figures and images for: Harnessing natural variation to identify cis regulators of sex-biased gene expression in a multi-strain mouse liver model
Source: PLoS Genet. 2021 Nov 9;17(11):e1009588. doi: 10.1371/journal.pgen.1009588 (PMC8664386; doi:10.1371/journal.pgen.1009588)

**A**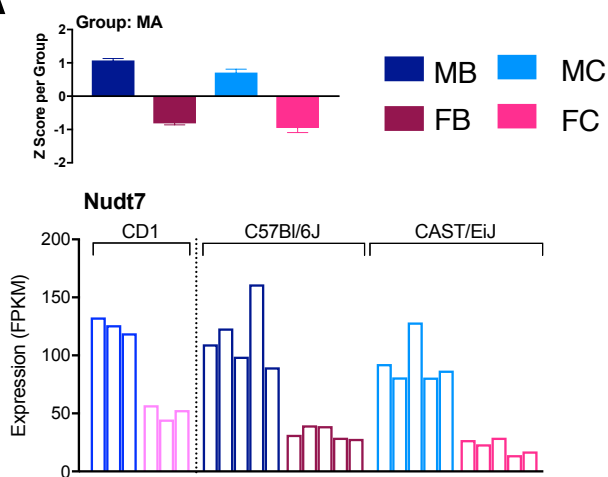**C**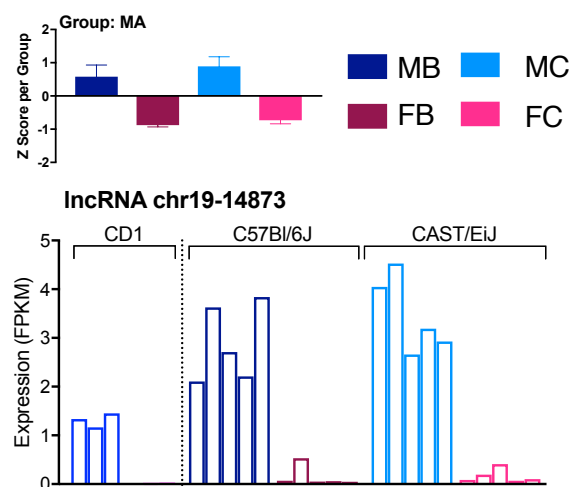**B**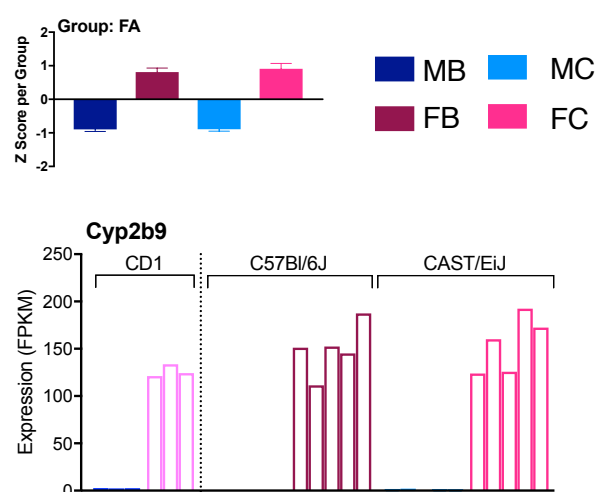**D**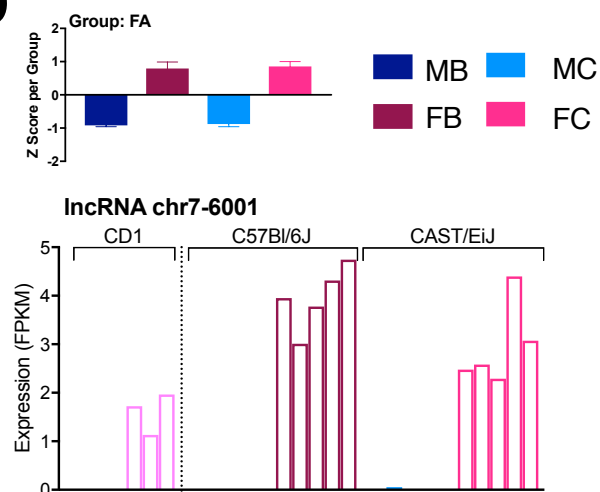**E** 144 strain-shared, sex-biased PC genes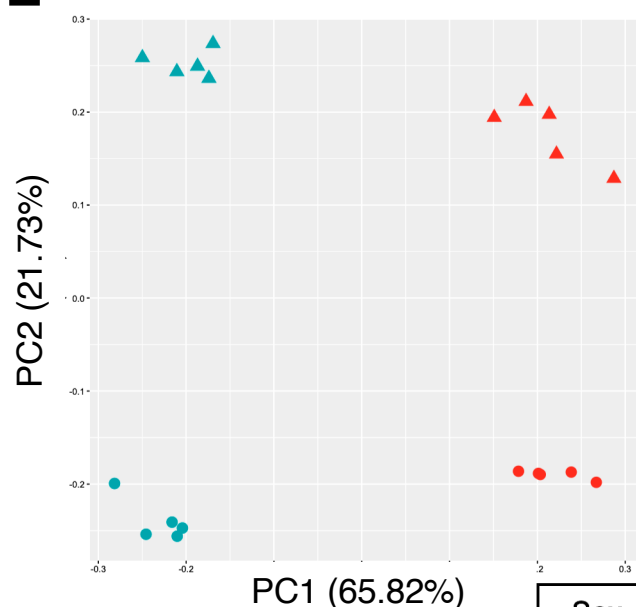**F** 78 strain-shared, sex-biased IncRNAs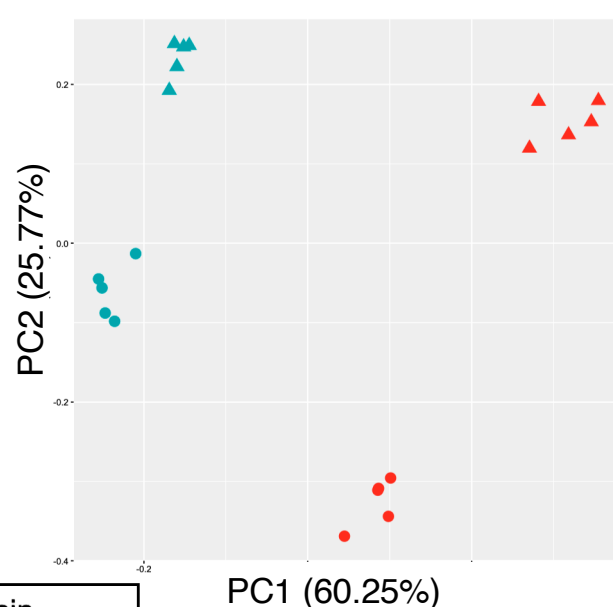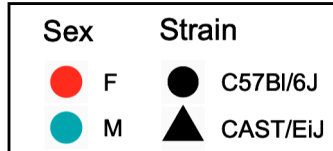

Supplement: S1 Fig — A-D. The top of each panel shows aggregated Z scores for cluster MA and FA indicated in Fig 1B (for protein-coding genes) and 1C (for lncRNA genes). The bottom panels show mouse liver expression data for the 4 indicated strain-conserved sex-biased genes, in FPKM units for 6 pooled CD-1 samples (n = 3 per sex), 10 individual B6 samples (n = 5 per sex), and 10 individual CAST samples (n = 5 per sex). C, D. Principal component analysis (PCA) for the 144 strain shared sex-biased protein coding genes (C) and for the 78 strain shared sex-biased lncRNA genes (D) reveals separation by sex along PC1 and by strain along PC2. The variance along each principal component is shown on each axis. (PDF) [file pgen.1009588.s001.pdf]

**A.** 289 B6-unique sex-biased lncRNAs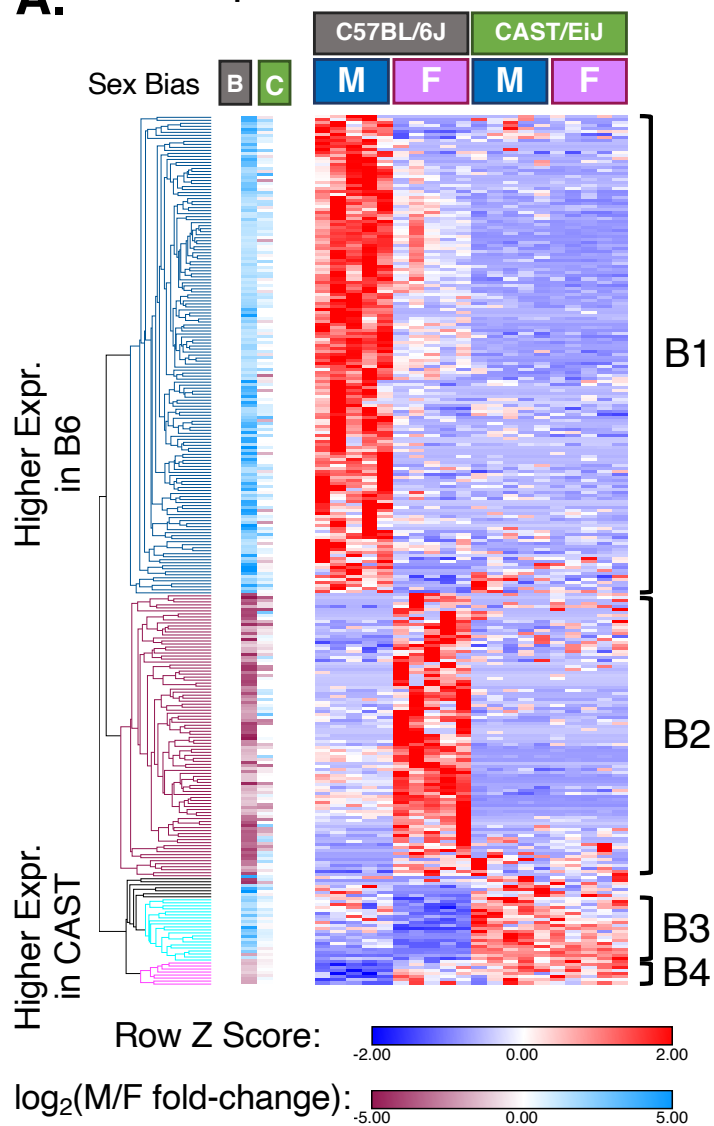**B.** 187 CAST-unique sex-biased lncRNAs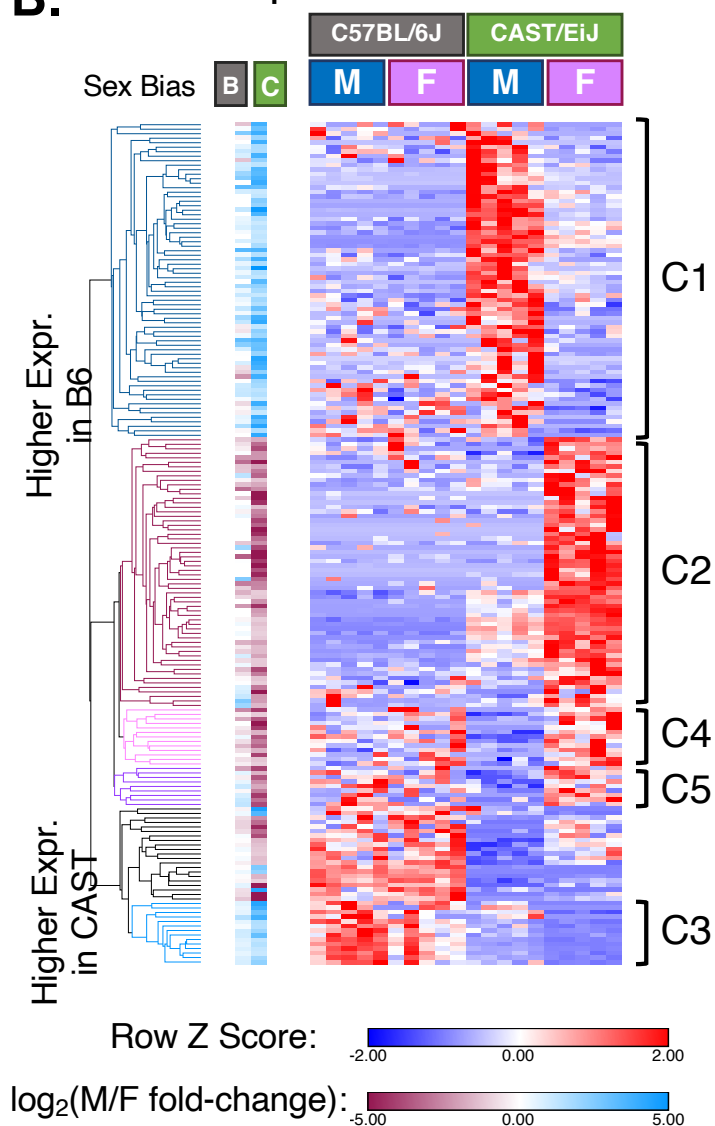

Supplement: S3 Fig — Heat maps presenting relative expression levels across individual mouse livers (n = 20; 5 per sex in each strain) for 289 B6-unique sex-biased lncRNAs (A) and for 187 CAST-unique sex-biased lncRNAs (B), based on data in Sheets E and F in S1 Table. Formatting and presentation are as described in S2 Fig. In A, clusters B1 and B2 comprise 253 genes that show higher expression in B6 mouse liver, and in B, clusters C1, C2, C4, C5 comprise 152 genes that show higher expression in CAST mouse liver. The reduced number of CAST-specific sex-biased lncRNAs could be a reflection of more rapid evolution of non-coding genes, but also is likely a due to the inclusion of B6 but not CAST mice in the datasets used for discovery and annotation of liver-expressed lncRNA genes [1, 2]. (PDF) [file pgen.1009588.s003.pdf]

**A.** B6  $\Delta$ K27ac (2,288 total)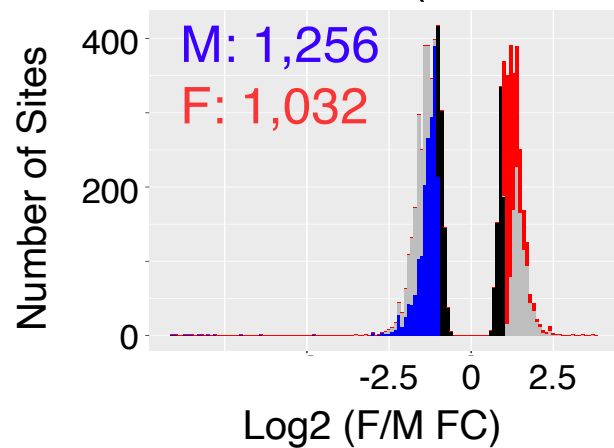**B.** CAST  $\Delta$ K27ac (2,571 total)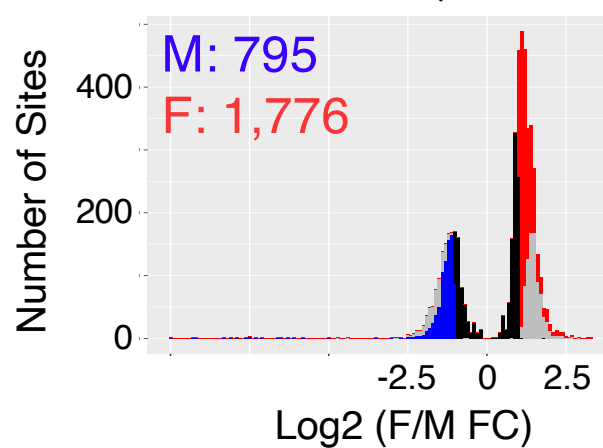**C.** B6  $\Delta$ DHS (1,311 total)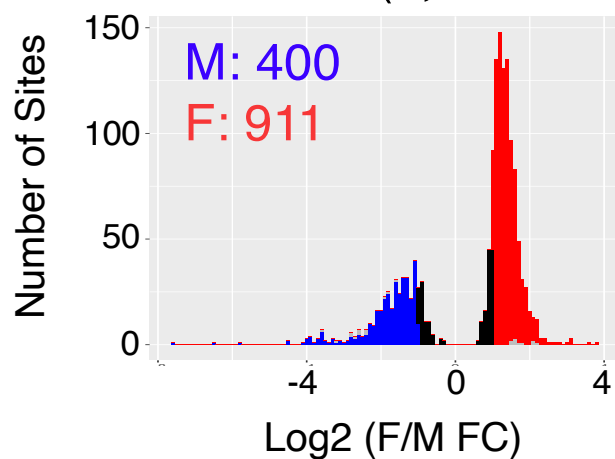**D.** CAST  $\Delta$ DHS (986 total)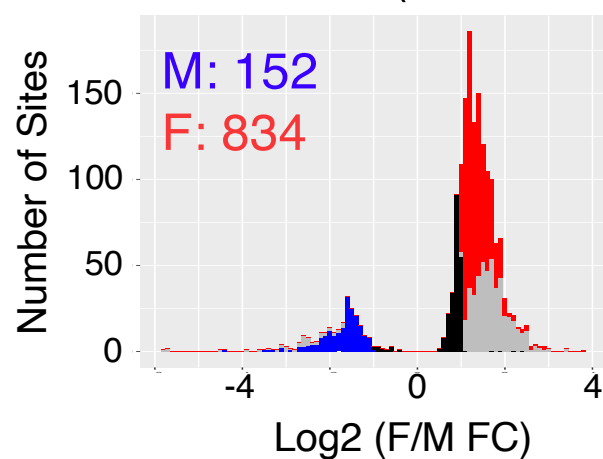

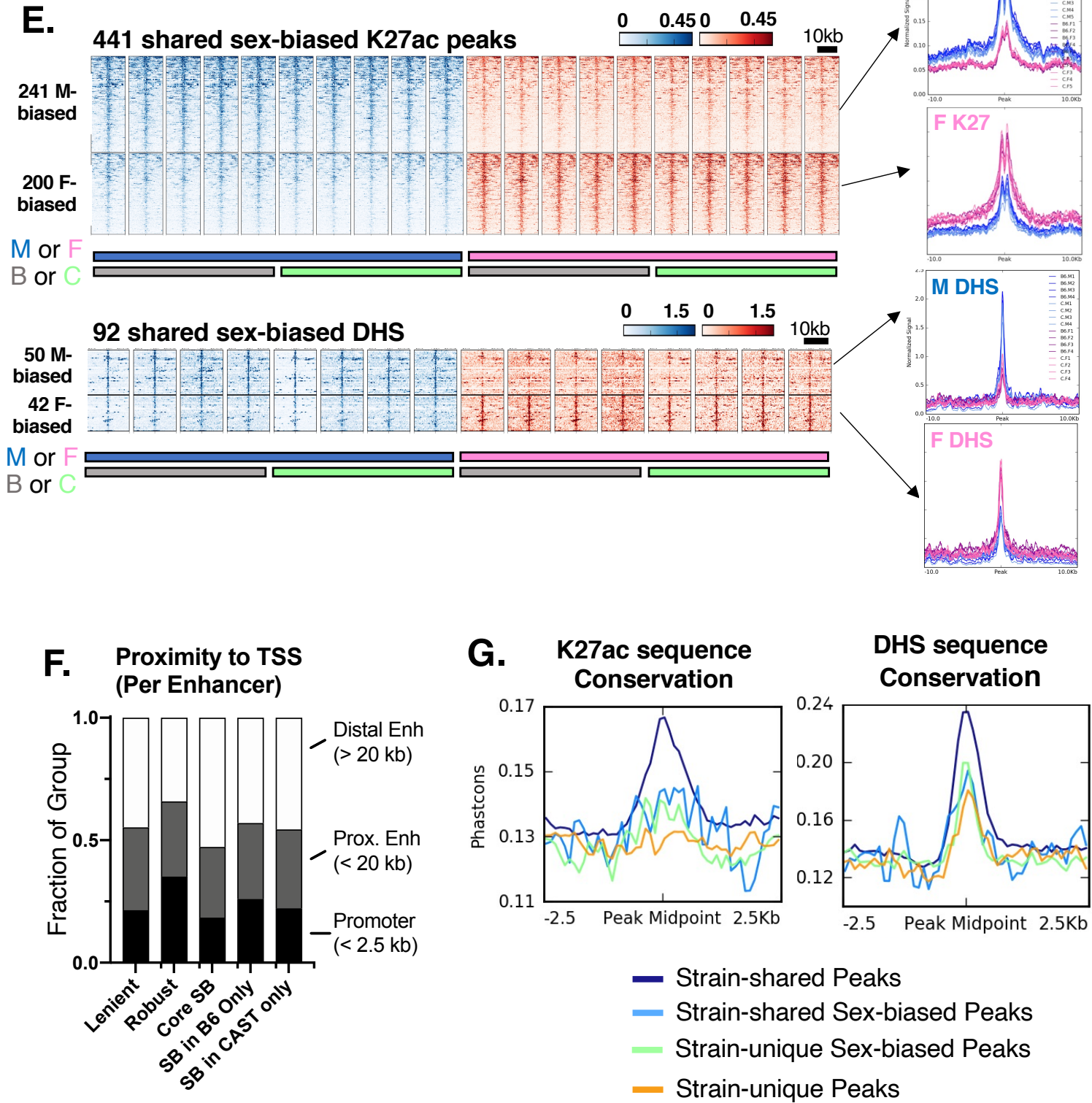

Supplement: S6 Fig — A. Number of differential H3K27ac peaks between male and female B6 mouse liver (y-axis) binned by the magnitude of fold-change in sex bias (log2 F/M as calculated by diffReps; x-axis). Shown are those sites that overlap MACS2 peaks. Blue bars indicate male-biased sites, red bars indicate female-biased sites, black bars indicate sites below the 2-fold fold-change cutoff (either direction), and gray bars indicate sites with low read count (minimum 15 reads in up regulated condition). In B6 mouse liver, differential analysis of in-peak diffReps sites identified 2,288 sex-biased H3K27ac peaks, of which 1,256 were male-biased and 1,032 were female-biased. To be considered sex-biased, the M/F or F/M fold-change > 2 with an FDR < 0.05 (n = 5 per sex; see Methods). B. Shown is the number of differential H3K27ac sites between male and female CAST mouse liver, as described for panel A. In CAST mouse liver, differential analysis of in-peak diffReps sites identified 2,571 sex-biased H3K27ac peaks, of which 795 were male-biased and 1,776 were female-biased. To be considered sex-biased, the M/F or F/M fold-change > 2 with an FDR < 0.05 (n = 5 per sex). Numbers differ slightly from Fig 2A due to small number of sites showing divergent sex bias between strains and in some cases a peak region identified in one strain that overlaps two peaks in one of the other strains in Fig 2A. C. Shown is the number of differential DHS between male and female B6 mouse liver (y-axis) binned by the magnitude of fold change sex bias (log2 F/M as calculated by diffReps; x-axis). Shown are those sites that overlap MACS2 peaks. Blue bars indicate male-biased sites, red bars indicate female-biased sites, black bars indicate sites below the 1.5-fold fold-change cutoff (either direction), and gray bars indicate sites with low read count (minimum 10 reads in upregulated condition). In B6 mouse liver, differential analysis of in-peak diffReps sites identified 1,311 sex-biased DHS, of which 400 were male-bias [file pgen.1009588.s006.pdf]

A.

*Gstp1*: M/F = 9.2 and 5.3

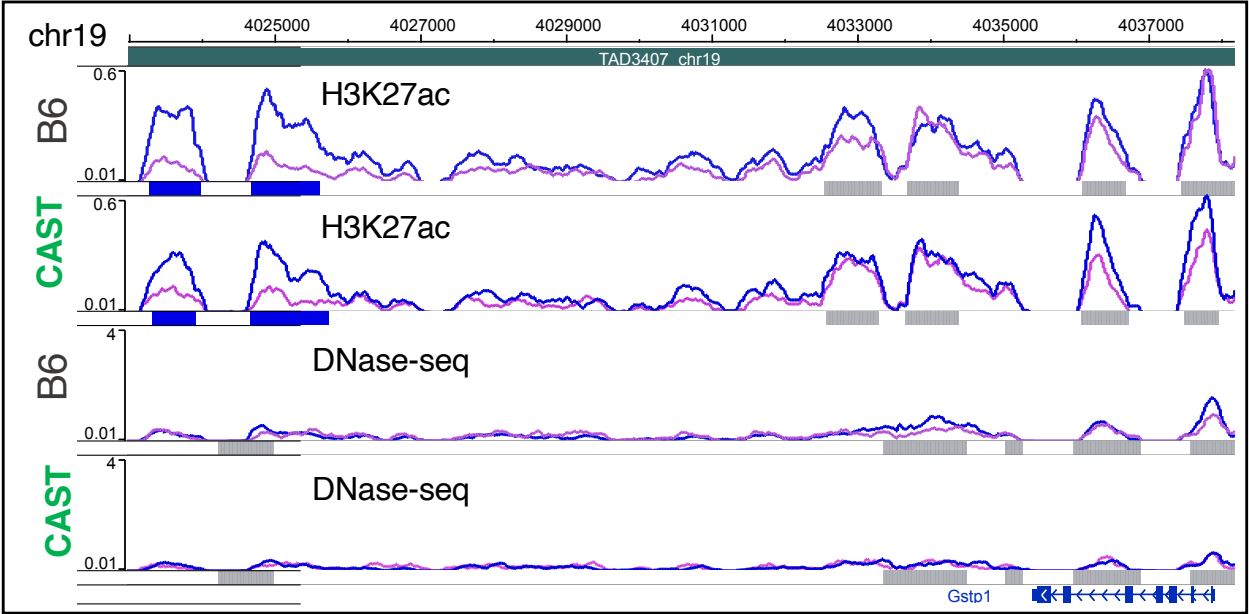

B.

*Elovl3*: M/F = 59.7 and 55.7

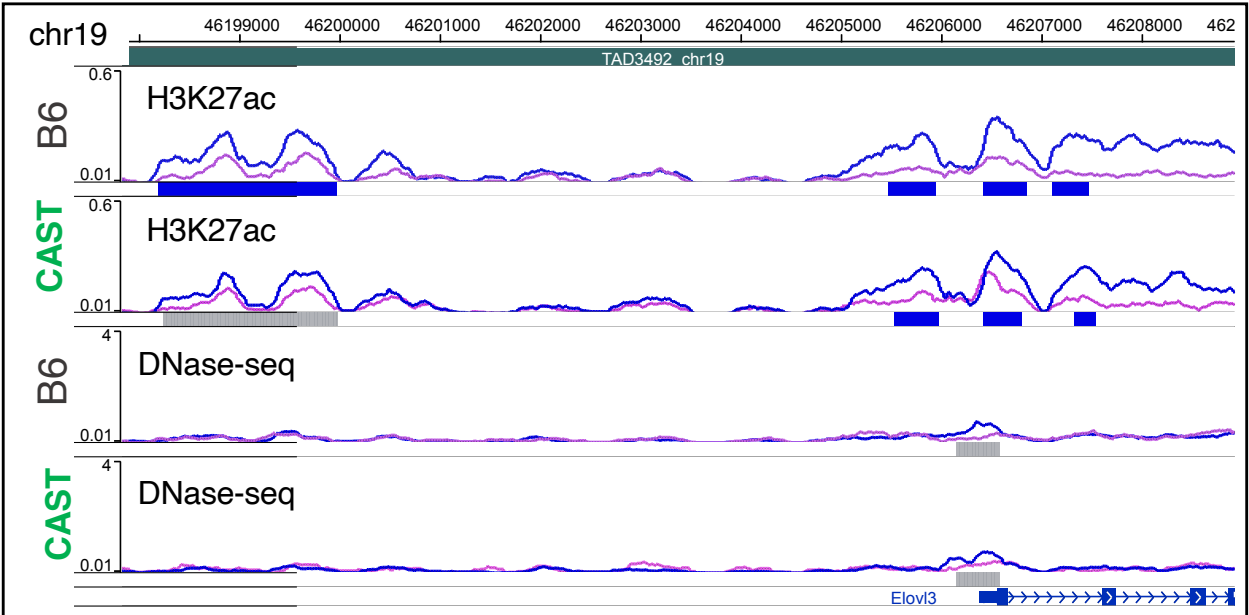

C.

*Nudt7*: IM/F = 3.5 and 4.0

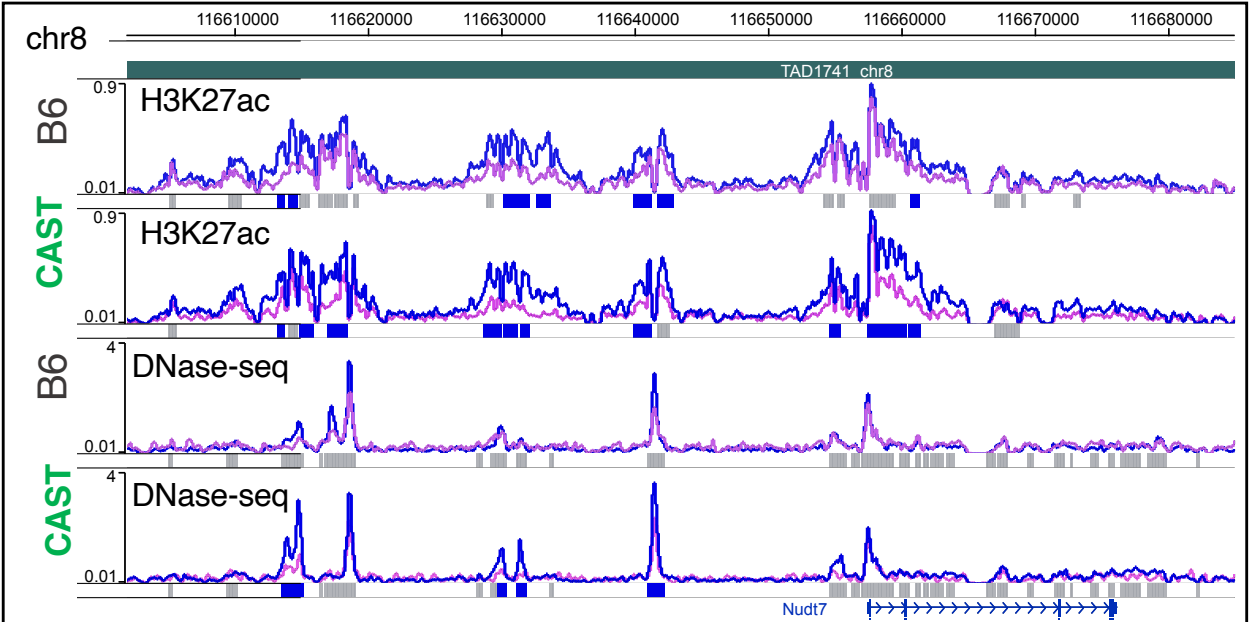

Supplement: S7 Fig — Annotations and formatting are as described in Fig 5. A. Gstp1 is a male-biased gene in both B6 and CAST mouse liver (M/F fold-change = 9.2 and 5.3, respectively). B. Elovl3 is a male-biased gene in both B6 and CAST mouse liver (M/F fold-change = 59.7 and 55.7, respectively). C. Nudt7 is a male-biased gene in both B6 and CAST mouse liver (M/F fold-change = 3.5 and 4.0, respectively). (PDF) [file pgen.1009588.s007.pdf]

**A.**

*Acot4*: F/M = 3.5 and 4.9  
*Acot3*: F/M = 34 and 69

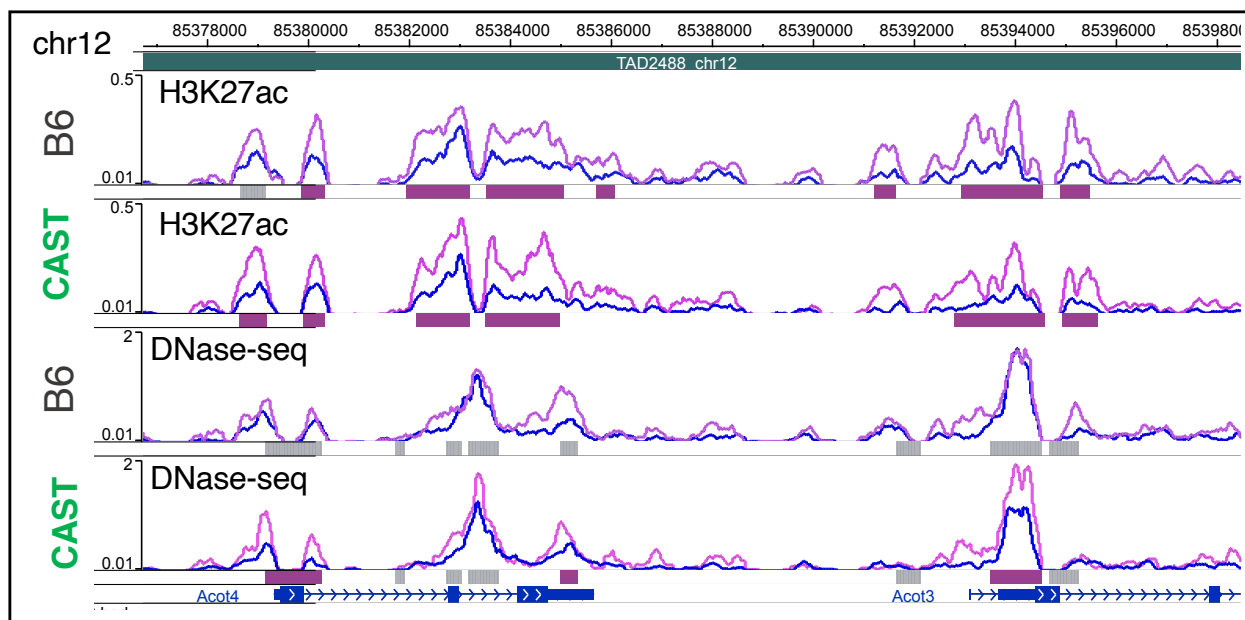**B.**

*Cyp4a14*: F/M = 18.4 and 416

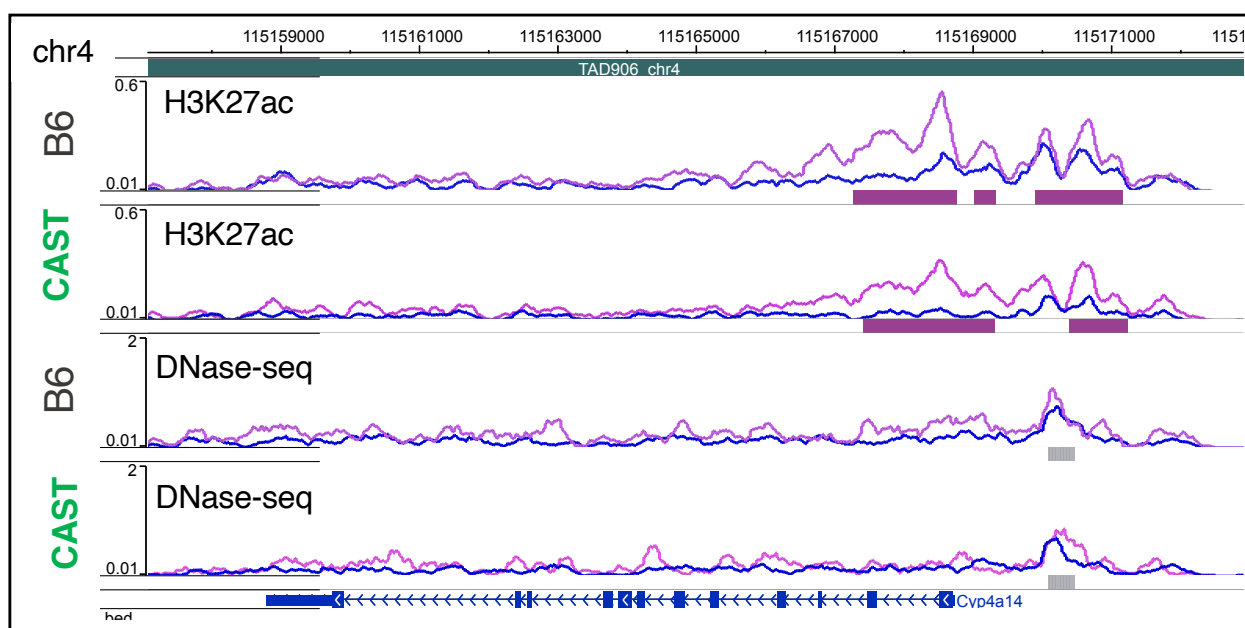**C.**

*Cux2*: F/M = 239 and 416

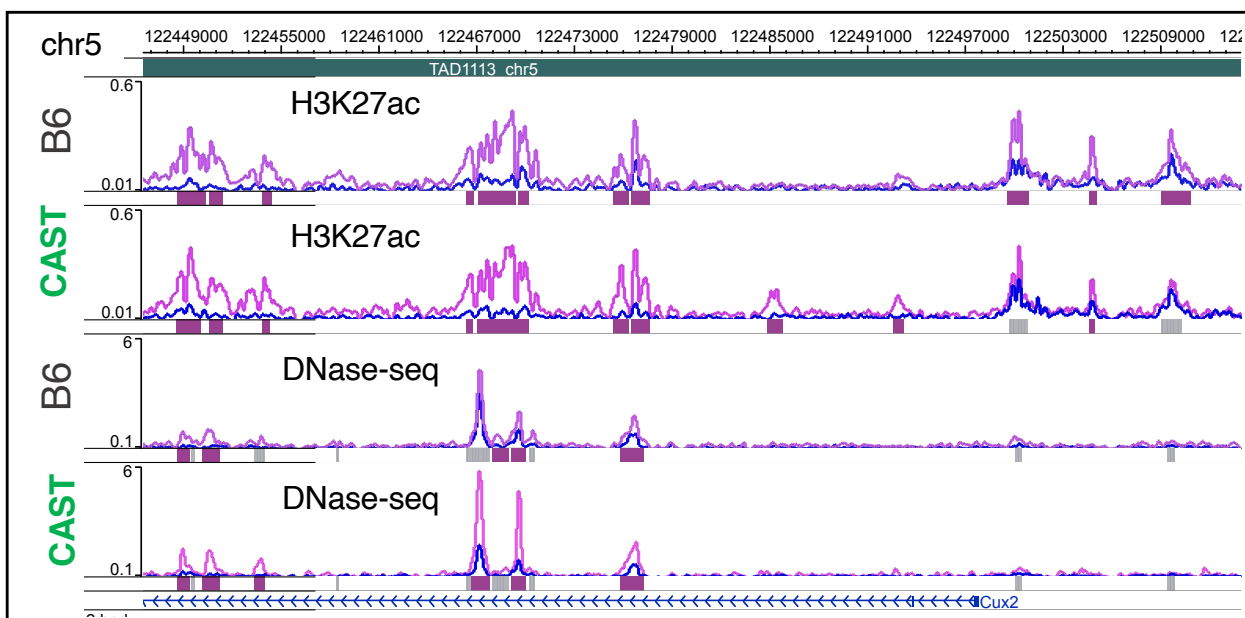

Supplement: S8 Fig — Annotations and formatting are as described in Fig 5. A. Acot3 and Acot4 are female-biased genes in both B6 and CAST mouse liver. Acot3 shows a greater magnitude of female-biased expression (F/M fold-change = 34 in B6 and 69 in CAST) compared to Acot4 (F/M fold-change = 3.5 in B6 and 4.9 in CAST). B. Cyp4a14 is a female-biased gene in both B6 and CAST mouse liver (F/M fold-change = 18.4 and 416, respectively). C. Cux2 is a female-biased gene in both B6 and CAST mouse liver (F/M fold-change = 239 and 416, respectively). (PDF) [file pgen.1009588.s008.pdf]

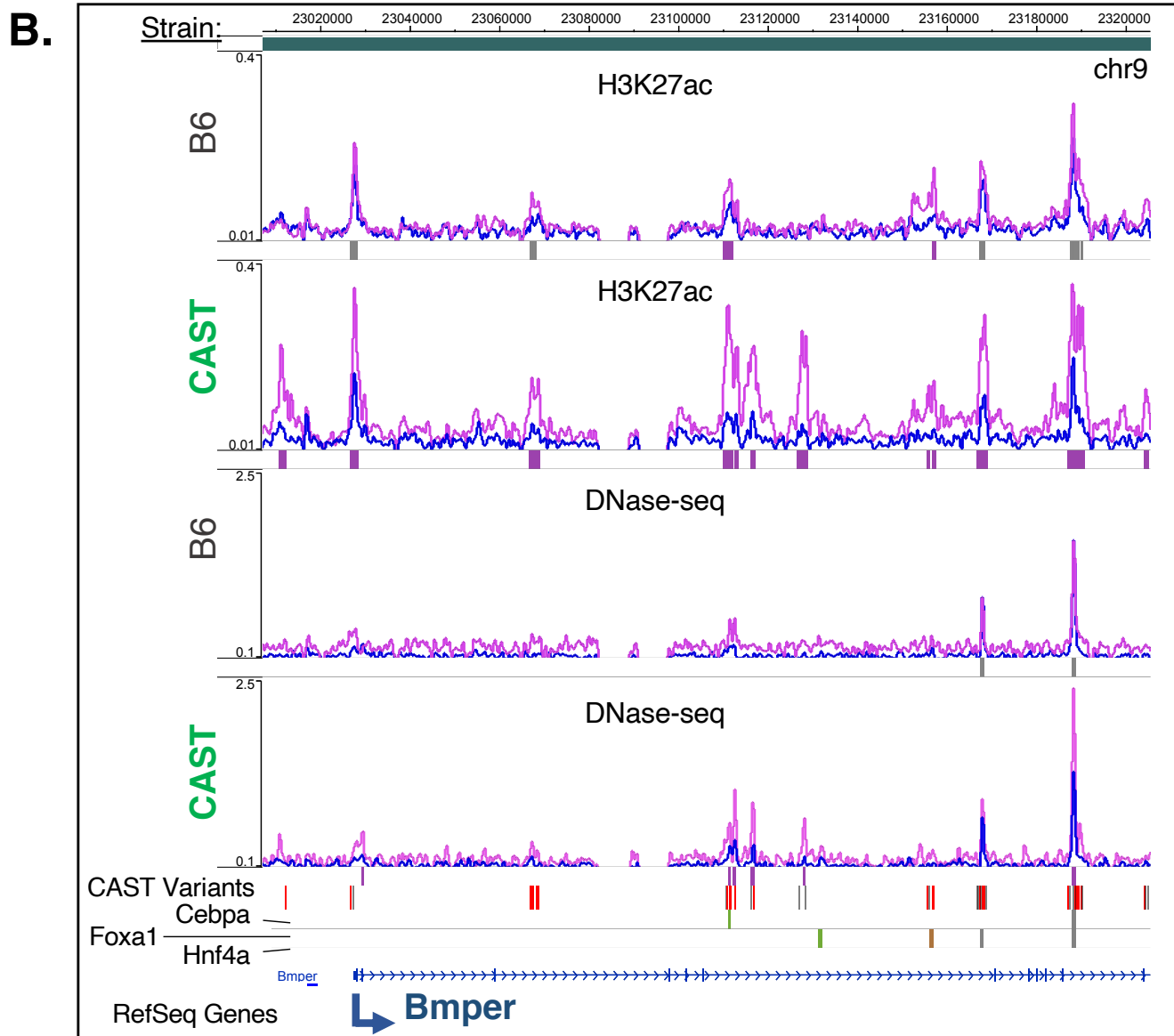

Supplement: S11 Fig — Annotations and formatting are as described for Fig 5. A. The female-biased gene Bmper is activated only in CAST female liver, resulting in a robust enhancement of female-biased expression in CAST mouse liver (significant only in CAST female; category #8 eQTL). This activation is associated with multiple genetic variants falling within several CREs, including both strain-shared male-biased peaks and CAST-unique male-biased peaks. CAST is the regulating strain only in female DO mouse liver, suggesting a strong sex-dependence of this effect (LOD = 18.69 in DO male livers with regression coefficient of +1.19). Given that there are two strain-shared (or “core”) female-biased enhancers, it is reasonable to conclude that one or both are sufficient to maintain female-biased expression (4.9-fold F/M in B6), while the additional sites are either additive or synergetic in further enhancing the expression and female bias in CAST liver (F/M fold-change of 17.4 in CAST). B. Shown is a WashU Epigenome Browser screenshot containing multiple CRE elements that gain female bias and activity in CAST liver in the genomic region neighboring the female-biased gene Bmper. Gray bars indicate single variants and red indicates multiple variants (2 or more) in the CAST Variants track. Additionally, three tracks indicate the strain-specificity of transcription factor binding (green indicates CAST-specific; orange indicates B6-preference) for three TFs: Cebpa, Foxa1, and Hnf4a (top to bottom). (PDF) [file pgen.1009588.s011.pdf]
