# Supplementary material for: Harnessing natural variation to identify cis regulators of sex-biased gene expression in a multi-strain mouse liver model
Source: PLoS Genet. 2021 Nov 9;17(11):e1009588. doi: 10.1371/journal.pgen.1009588 (PMC8664386; doi:10.1371/journal.pgen.1009588)
Supplement: S2 Fig — Heat maps presenting relative expression levels across individual mouse livers (n = 20; 5 per sex in each strain) for 301 B6-unique sex-biased protein-coding genes (A) and for 207 CAST unique sex-biased protein-coding genes (B), based on data in Sheets C and D in S1 Table. Expression values are shown as Z-scores normalized per row to visualize patterns independent of the expression level. Log2 (M/F fold-change) values are shown at the left, with blue indicating male bias and purple indicating female bias for B6 (column marked ’B’) and CAST datasets (column marked ’C’). Hierarchical clustering was performed based on Euclidean distance and is shown to the left of each heat map, with colors indicating the cluster identity labelled to the right of the heat map. In A, clusters B1 and B2 comprise 238 genes that show higher expression in B6 mouse liver; and in B, clusters C1 and C2 comprise 142 genes that show higher expression in CAST mouse liver. (PDF) [file pgen.1009588.s002.pdf]

S2 Fig

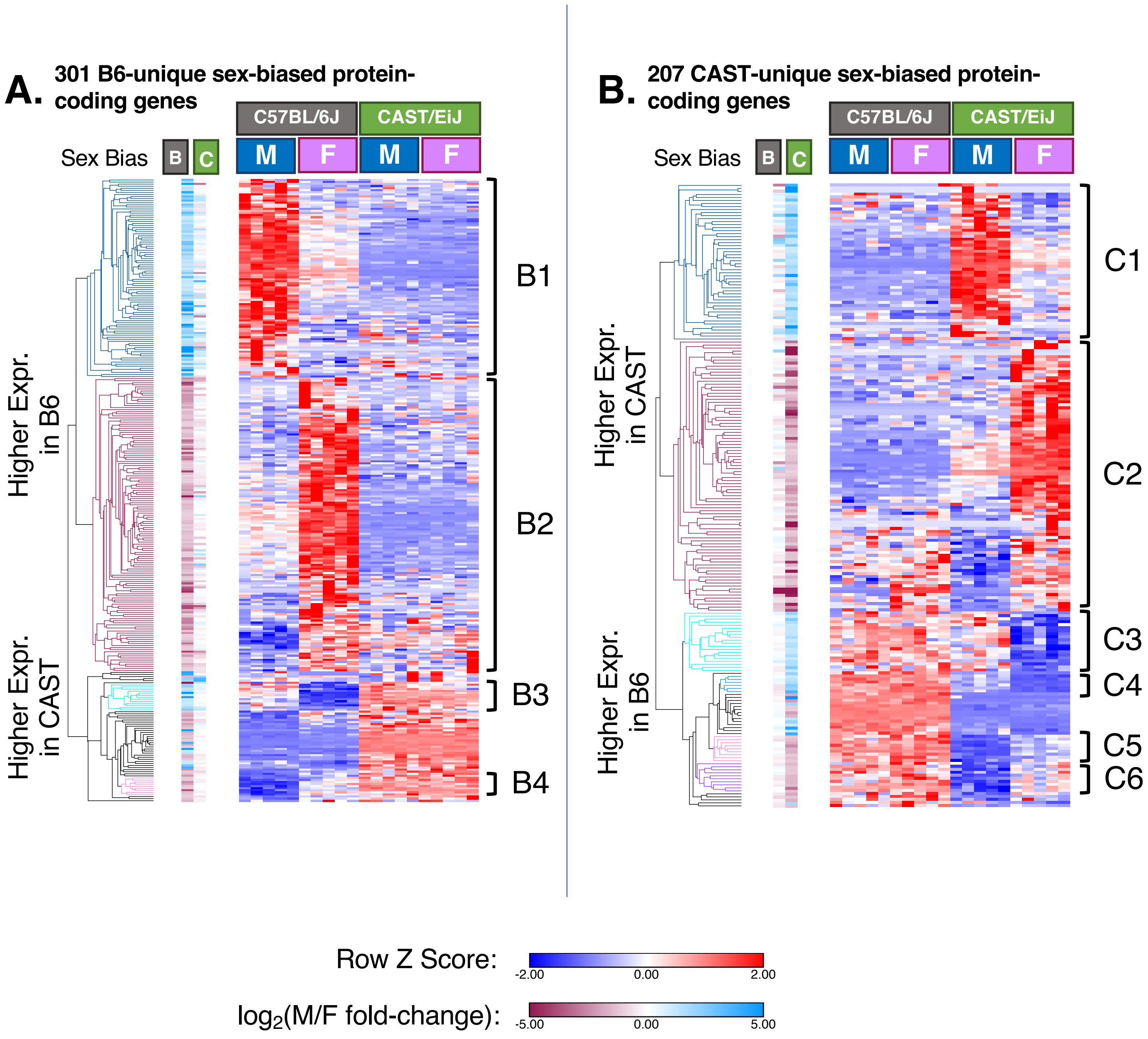

A.
301 B6-unique sex-biased protein-coding genes
C57BL/6J
CAST/EiJ
Sex Bias
B
C
M
F
M
F
Higher Expr. in B6
Higher Expr. in CAST
B1
B2
B3
B4
B.
207 CAST-unique sex-biased protein-coding genes
C57BL/6J
CAST/EiJ
Sex Bias
B
C
M
F
M
F
Higher Expr. in CAST
Higher Expr. in B6
C1
C2
C3
C4
C5
C6
Row Z Score:
-2.00
0.00
2.00
log2(M/F fold-change):
-5.00
0.00
5.00
