# Supplementary material for: Harnessing natural variation to identify cis regulators of sex-biased gene expression in a multi-strain mouse liver model
Source: PLoS Genet. 2021 Nov 9;17(11):e1009588. doi: 10.1371/journal.pgen.1009588 (PMC8664386; doi:10.1371/journal.pgen.1009588)
Supplement: S4 Fig — Expression data are presented as FPKM values for 6 pooled CD-1 livers (n = 3 per sex), 10 individual B6 livers (n = 5 per sex), and 10 individual CAST livers (n = 5 per sex). A. Three transcriptional regulators with well-established roles in liver sexual dimorphism (Stat5b, Foxa2, and Hnf4a). No sex-dependence or strain bias is found. B. Three transcriptional regulators that are expressed in a sex-dependent manner, and in the case of Cux2 and Bcl6, have established roles in the regulation of liver sexual dimorphism. C. Examples of sex-biased TF genes from S2A Fig, clusters B2, B3 and B4. These genes are putative transcriptional regulators presented in S3 Table. The three B6-unique sex-biased TFs also show sex-biased expression in CD-1 livers (fold-change > 1.5 and FDR < 0.05). D. Examples of sex-biased TF genes from S2B Fig, clusters C3 and C5. E. Shown is the total number of liver sex-biased protein-coding (PC) genes (top) and lncRNA genes (bottom) across B6, CAST, and CD-1 mice. The impact of increasing the threshold for sex bias from standard (2-fold) to 4-fold, both at FDR< 0.05, is also shown. From left to right, the number of sex-biased genes at the specified threshold is shown for the following groups: PolyA+ RNA-seq from B6 and CAST (this study), and from CD-1 mouse liver [3], B6 microarray (Jax Strain Survey 26), and CAST microarray (Jax Strain Survey 26; http://cgd.jax.org/gem/strainsurvey26/v1). A total of 852, 661, and 826 standard sex-biased genes were identified in B6, CAST, and CD-1 mice, respectively (protein coding and lncRNA genes combined). Similarly, a total of 391, 313, and 389 strictly sex-biased genes were identified in each strain, respectively. (PDF) [file pgen.1009588.s004.pdf]

S4A, S4B Fig

# Strain-conserved TFs

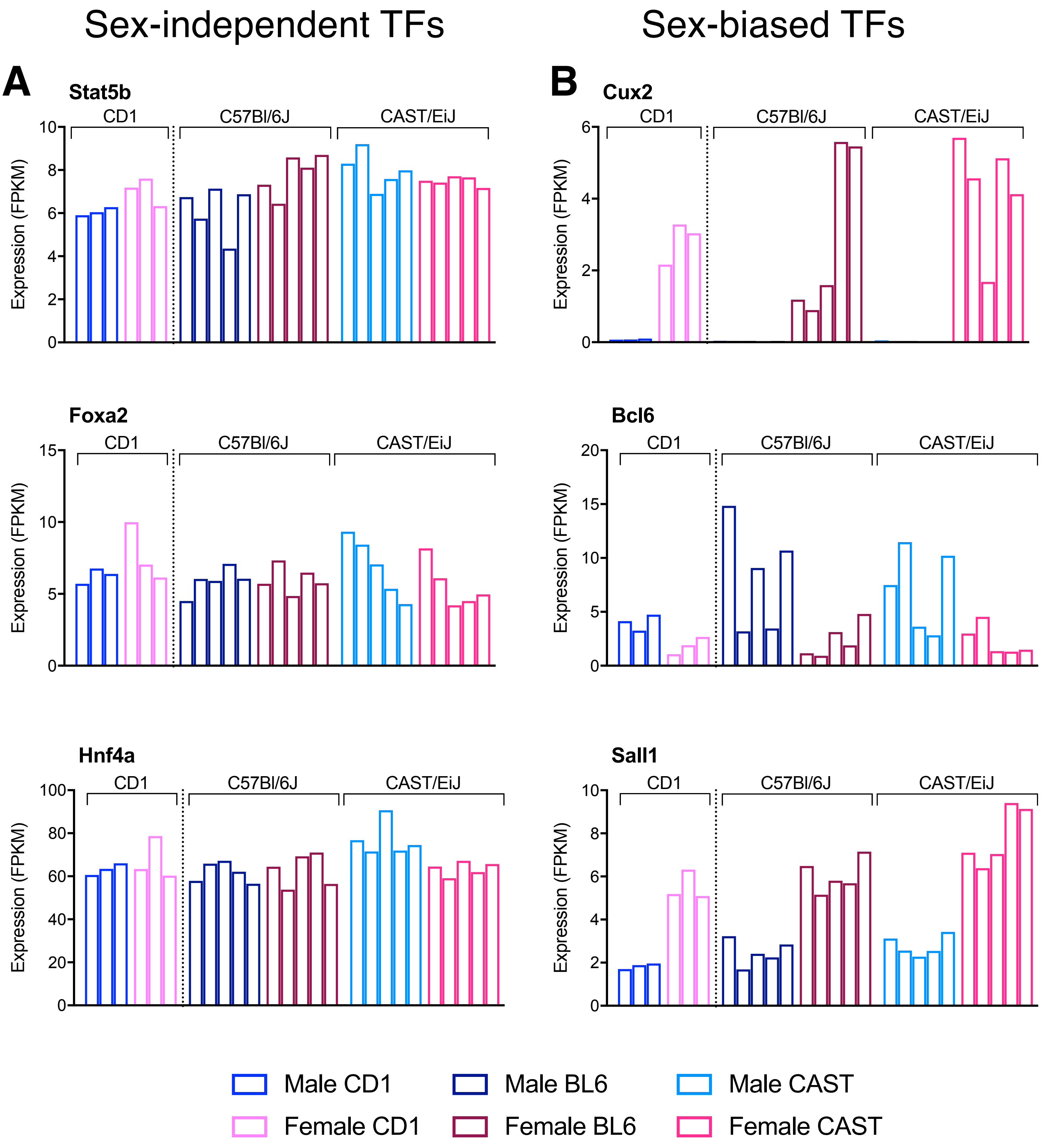

S4C-S4E Fig

# Strain-dependent TFs

C

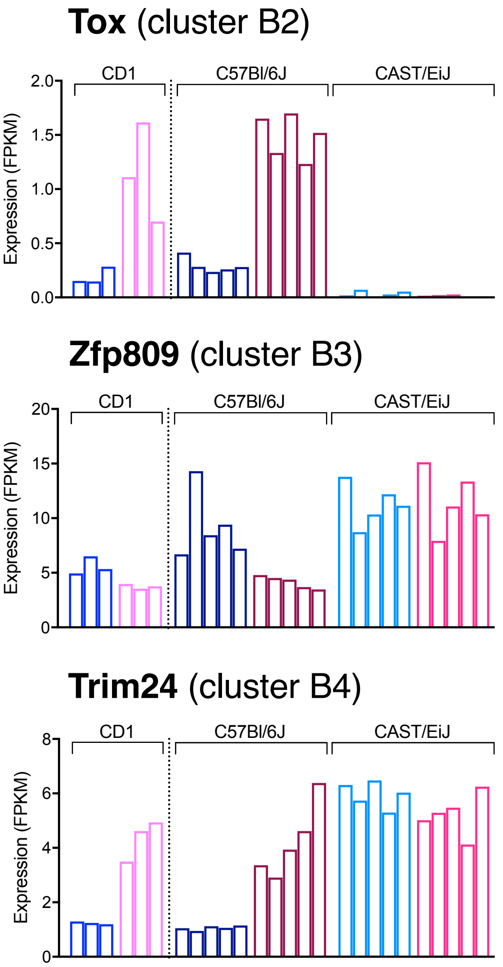


D

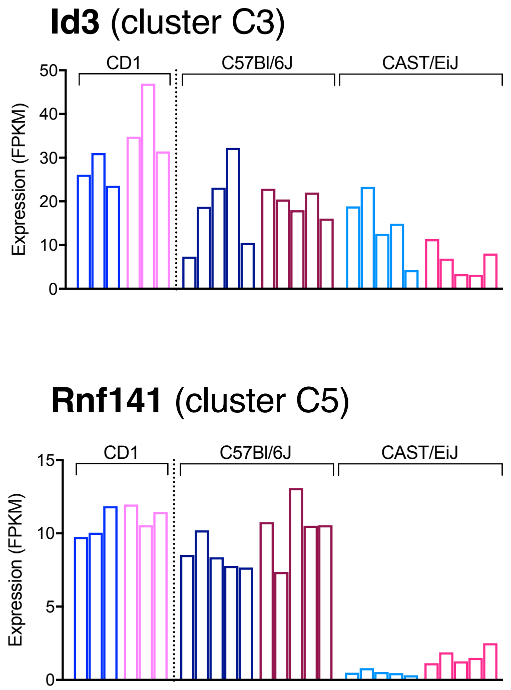


E

| | *Number of genes* | | | Microarray (3) | |
|---|---|---|---|---|---|
| PC-genes (excl. chrY) | **C57BL/6J** (1) | **CAST/EiJ** (1) | **CD1** (2) | **C57BL/6J** | **CAST/EiJ** |
| Sex-biased (standard) | 480 | 392 | 490 | 157 | 104 |
| Sex-biased (strict; FC > 4) | 183 | 149 | 192 | 18 | 14 |

| lncRNAs (excl. chrY) | **C57BL/6J** (1) | **CAST/EiJ** (1) | **CD1** (2) | |
|---|---|---|---|---|
| Sex-biased (standard) | 372 | 269 | 336 | (1) This study<br>(2) GSE98586<br>(3) Jax strainsurvey |
| Sex-biased (strict; FC > 4) | 208 | 164 | 197 | |
