# Supplementary material for: Harnessing natural variation to identify cis regulators of sex-biased gene expression in a multi-strain mouse liver model
Source: PLoS Genet. 2021 Nov 9;17(11):e1009588. doi: 10.1371/journal.pgen.1009588 (PMC8664386; doi:10.1371/journal.pgen.1009588)
Supplement: S5 Fig — We used qPCR to interrogated known sex-biased enhancers, identified in CD-1 mouse liver (browser screen shots to the right of each panel), within 15 kb of the female-biased gene Cyp3a16 (A), and the male-biased genes Cyp7b1 (B) and Gstp1 (C). All three genes have nearby CAST-specific TF binding sites, as identified in male mouse liver [4]; these strain-specific sites are statistically significant in all cases but with a magnitude of strain-preference varies, ranging up to >100-fold CAST-specific, as marked in the panels on the right side of each figure. Expression values (in FPKM) are from S1 Table and are based on RNA-seq using male B6 (cluster MB; n = 5), female B6 (cluster FB; n = 5), male CAST (cluster MC; n = 5), and female CAST (cluster FC; n = 5) mouse liver. The magnitude of sex bias calculated by EdgeR is shown above each pair of gray and green bars (linear M/F values). Genomic locations of the qPCR amplicons used to interrogate the DHS and H3K27ac-ChIp’d DNA are indicated in each browser panel, with primer sequences shown in Sheet A in S4 Table. Strain-specific TF binding sites are listed in S6 Table. DNase-qPCR results (set of 4 bars at the middle of each panel) are presented as the signal in the indicated genomic region divided by the average of 3 negative control regions (see Methods). A. Female-biased chromatin opening for a DHS on chromosome 5 near Cyp3a16 is seen in CD-1 and CAST but not B6 mouse liver. For the enhancer neighboring Cyp3a16, female-biased chromatin opening is seen in CAST mouse liver (*, p = 0.0159) but not B6 mouse liver. Further, greater chromatin opening is seen in CAST relative to B6 (male B6 vs male CAST, * p = 0.0168; and female B6 vs female CAST liver, ** p = 0.0072; t-test). This strain difference in chromatin opening could be explained by CAST-specific binding of the TF Foxa1 at this genomic region, even though Cyp3a16 is not differentially expressed between the strains. The WashU Epigenome Browser screenshot (right) shows th [file pgen.1009588.s005.pdf]

S5 Fig

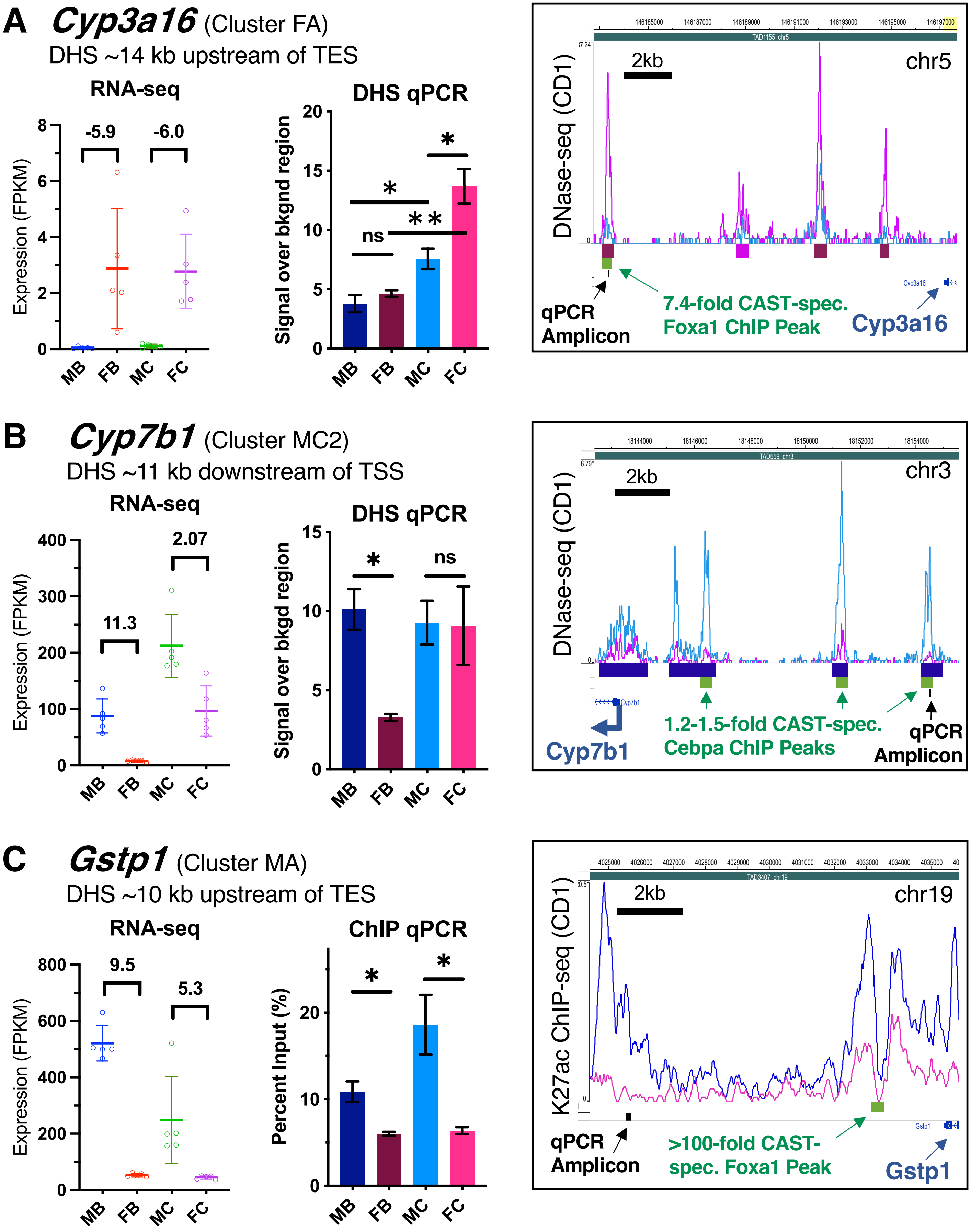
A
Cyp3a16 (Cluster FA)
DHS ~14 kb upstream of TES
RNA-seq
Expression (FPKM)
-5.9
-6.0
MB
FB
MC
FC
DHS qPCR
Signal over bkgnd region
ns
*
* *
DNase-seq (CD1)
2kb
chr5
qPCR
Amplicon
7.4-fold CAST-spec.
Foxa1 ChIP Peak
Cyp3a16
B
Cyp7b1 (Cluster MC2)
DHS ~11 kb downstream of TSS
RNA-seq
Expression (FPKM)
11.3
2.07
DHS qPCR
Signal over bkgd region
*
ns
DNase-seq (CD1)
2kb
chr3
Cyp7b1
1.2-1.5-fold CAST-spec.
Cebpa ChIP Peaks
qPCR
Amplicon
C
Gstp1 (Cluster MA)
DHS ~10 kb upstream of TES
RNA-seq
Expression (FPKM)
9.5
5.3
ChIP qPCR
Percent Input (%)
*
K27ac ChIP-seq (CD1)
2kb
chr19
qPCR
Amplicon
>100-fold CAST-
spec. Foxa1 Peak
Gstp1
