# Supplementary material for: Harnessing natural variation to identify cis regulators of sex-biased gene expression in a multi-strain mouse liver model
Source: PLoS Genet. 2021 Nov 9;17(11):e1009588. doi: 10.1371/journal.pgen.1009588 (PMC8664386; doi:10.1371/journal.pgen.1009588)
Supplement: S9 Fig — A. 491 eQTLs have CAST or B6 as the regulating strain in at least 1 of 3 comparisons: all DO mouse liver samples, DO male liver samples only, and DO female liver samples only. We identified 406 eQTLs for which CAST is the regulating strain and 85 for which B6 is the regulating strain. There are 7 possible combinations based on significant in all liver samples (A), significant in male liver samples (M), and significant in female liver samples (F), as indicated below the x-axis. The distribution of eQTLs in each of these 7 groups (per strain) is shown. B. Left: Boxplots indicating the absolute Log2 fold change of genes regulated by lenient eQTLs (blue) and genes regulated by robust eQTLs (red). The absolute fold change (M/F or F/M) of genes regulated by robust eQTLs is higher than those of genes regulated by lenient eQTLs (p = 6.12e-17, student’s T-test). Right: Boxplots indicating the maximum expression in Log2(FPKM+1) of genes regulated by lenient eQTLs (blue) and genes regulated by robust eQTLs (red). The median expression of genes regulated by robust eQTLs is higher, but this difference is not significant (p = 0.0831, student’s T-test). C. Left: Boxplots indicating the distribution of sizes of eQTLs for which B6 (median size 2.14 Mb; n = 406) or CAST (median size 1.7 Mb; n = 85) is the regulating strain. Right: Boxplots indicating the number of strain-specific variants in eQTLs for which B6 (median variant count 320; n = 85 eQTLs) or CAST (median variant count 6,299; n = 406 eQTLs) is the regulating strain. D. Flowchart depicting the filtering strategy for identifying strain-specific SNPs and Indels relevant for sex-biased gene expression. All variants specific to CAST or B6 mice across the 8 founder strains were filtered to consider only those that fall within the 491 eQTLs associated with sex-biased genes, and for which CAST or B6 is the regulating strain. Finally, variants falling within the TAD-eQTL overlap and within cis-regulatory elements are considered as [file pgen.1009588.s009.pdf]

S9A-S9D Fig

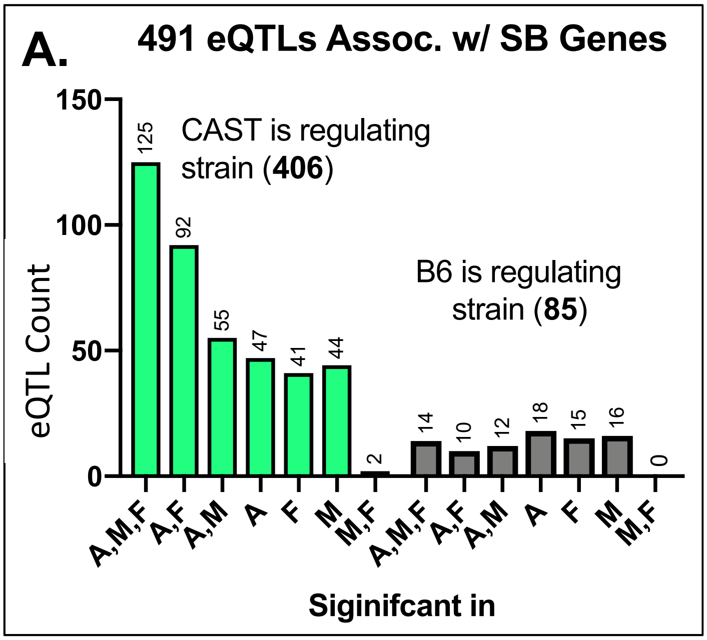

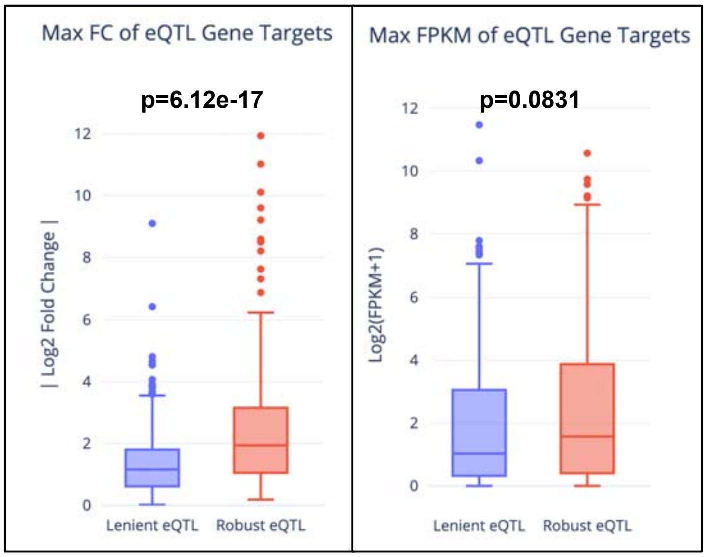

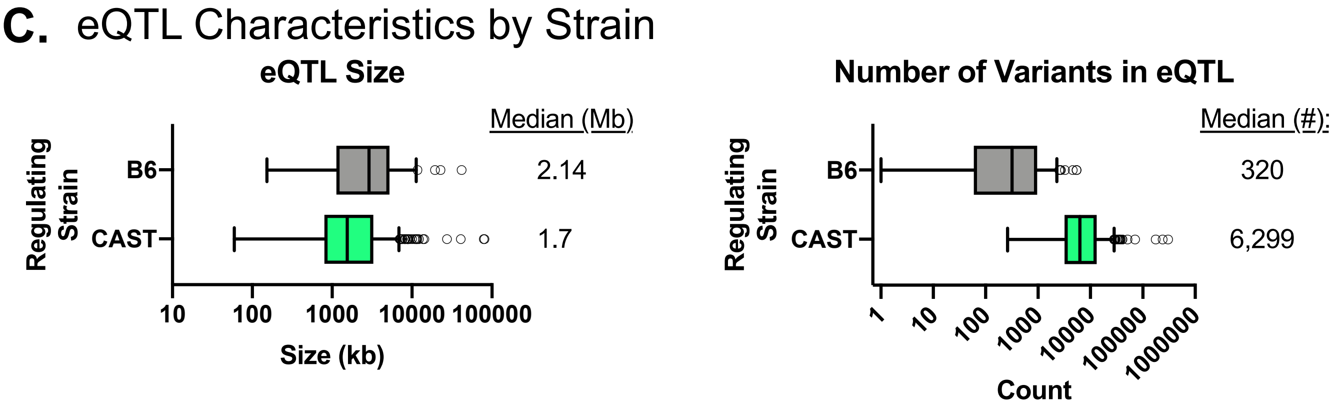

D.

Strain-specific from the regulating strain for all:

Total Variants Genome-wide
B6-specific or CAST-specific

SNPs: 8,442,305
Indels: 1,753,541
Total = 10,195,846

→

Variants within eQTLs
491 eQTLs for Sex-biased genes
2,864,366 SNPs
617,117 Indels
Total = 3,481,483

(1.7 Mb median size,
4.8 k variants per region)

↓

Variants within CREs
103,491 SNPs
20,715 Indels
Total = 124,206

→

Variants within TAD
for 383 TAD-overlapping eQTLs
594,477 SNPs
122,791 Indels
Total = 717,268

(0.42 Mb median size,
1.6 k variants per region)

↓

Variants within CREs
26,785 SNPs
5,265 Indels
Total = 32,050

S9EF Fig

E.

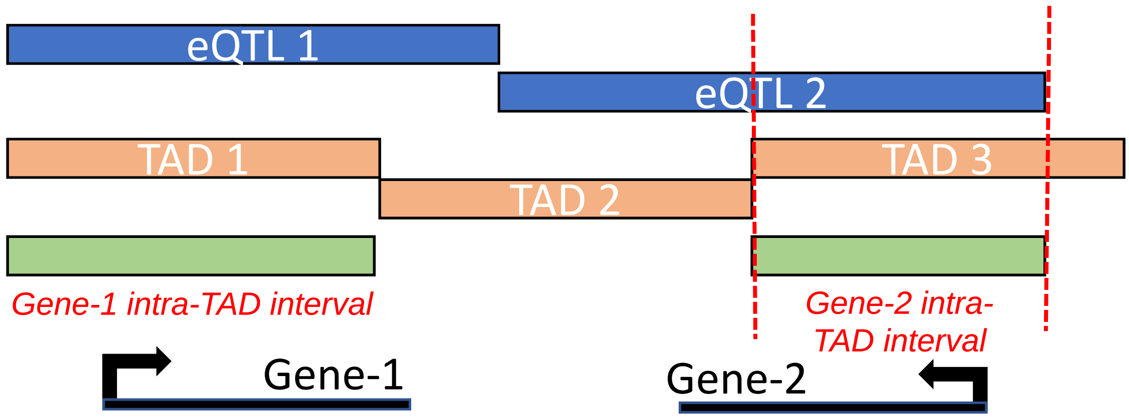

F. eQTL Interval Filtering

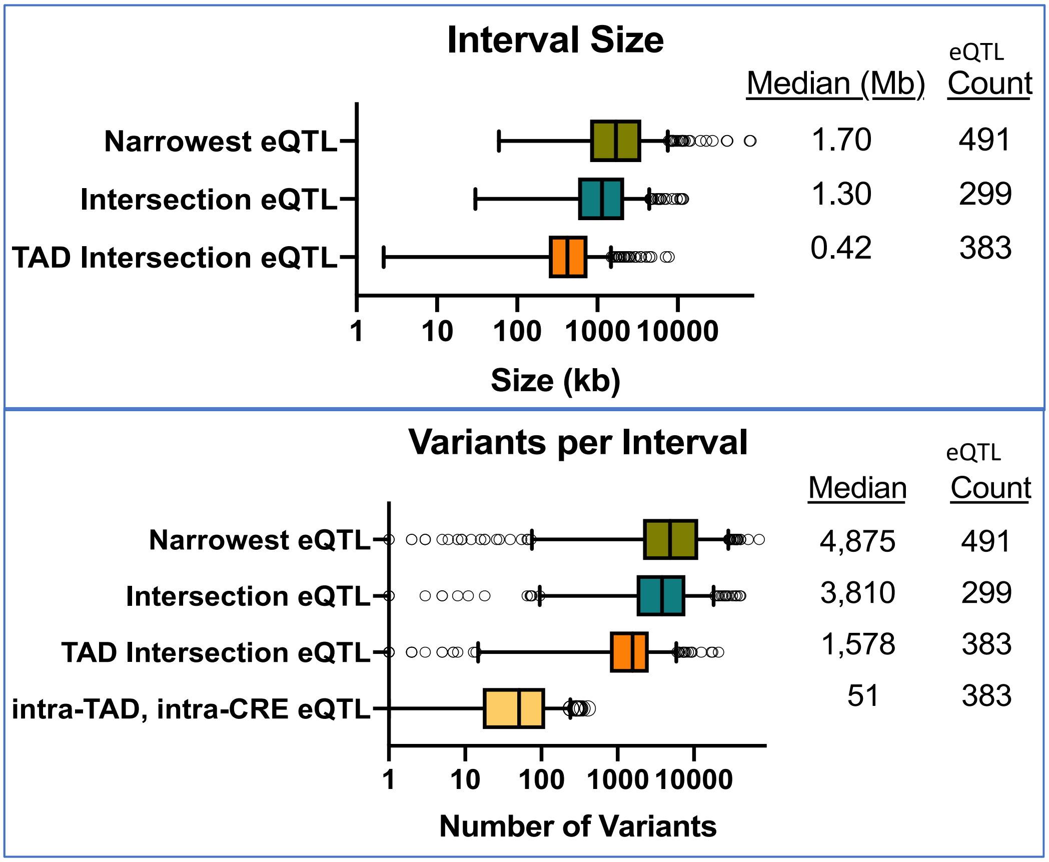

S9GH Fig

G. eQTL Categorization (see **Table S6G** for lists eQTL subsets)

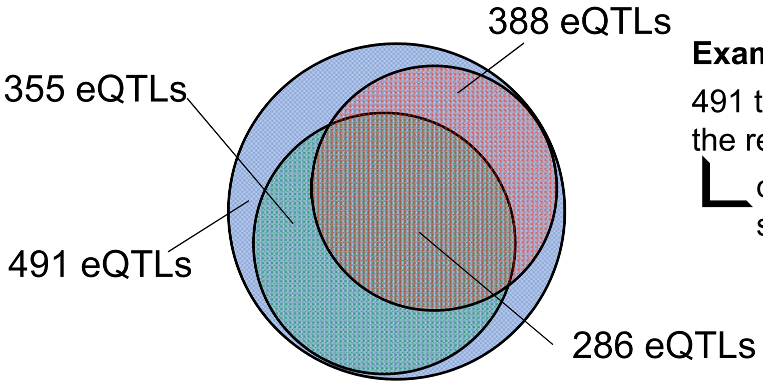

Example:

491 total eQTLs where CAST or B6 is the regulating strain

- of which 355 eQTLs regulate genes sex-biased in B6 and/or CAST liver
  - of which 286 fall into cat.# 1-8: 138 in cat.# 1-4, 148 in cat. #5-8.

491 eQTLs: Total number of eQTLs for which the regulating strain is CAST or B6 and the regulated gene is sex-biased in at least one DO founder strain
355 eQTLs: eQTLs for which the regulating strain is CAST or B6 and the regulated gene is sex-biased in B6 and/or CAST (sex bias based on RNA-seq, this study)
388 categorized eQTLs: eQTLs for which the regulating strain is CAST or B6, the regulated gene is sex-biased in at least one DO founder strain, the eQTL is stronger in male than female DO mice (by LOD score), or vice versa, and CAST or B6 is the regulating strain for the cohort (male-only or female-only DO) with the stronger effect.
286 eQTLs: Intersection of the set of 355 eQTLs regulating genes showing sex-bias in CAST and/or B6 mouse liver with the set of 388 categorized eQTLs, described above.

H. eQTL Categorization, cont'd. (see **Table S6G**)

388 categorized eQTLs for which the regulating strain is CAST or B6

286 eQTLs whose genes show sex-bias in CAST and/or B6 liver

166 eQTLs with sex-biased CREs gained or lost in CAST or B6 liver
Of these: 61 have a single relevant CRE gain/loss containing variants, and 105 have multiple CREs gained/lost containing variants)

208 eQTLs resulting in either a complete gain, loss, or reversal of sex bias in CAST or B6 liver

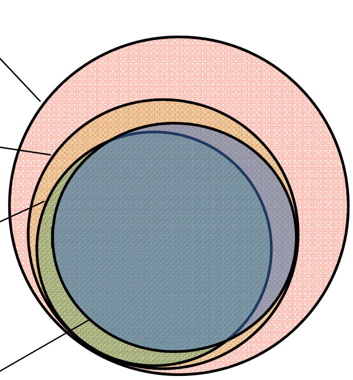

Example:

388 total categorized eQTLs where CAST or B6 is the regulating strain

- of which 286 fall into cat.# 1-8: 138 in cat.# 1-4, 148 in cat. #5-8
  - of which 166 contain a minimum of one sex-biased CRE gained/lost
    - of which 61 have a single relevant CRE gain/loss containing variants, and 105 have multiple CREs gained/lost containing variants
