# Supplementary material for: Harnessing natural variation to identify cis regulators of sex-biased gene expression in a multi-strain mouse liver model
Source: PLoS Genet. 2021 Nov 9;17(11):e1009588. doi: 10.1371/journal.pgen.1009588 (PMC8664386; doi:10.1371/journal.pgen.1009588)
Supplement: S10 Fig — Annotations and formatting are as described in Fig 5. A. The male-biased gene Olfm2 is repressed in CAST male liver, resulting in a loss of sex-biased expression in CAST mice (category #1 eQTL). This repression is associated with multiple genetic variants falling within a male-biased peak that is lost in CAST mice (this is the only such region in the eQTL; see Sheet A in S6 Table). Two of these variants show substantial binding preference for B6 over CAST for the TFs Cebpa (16-fold) and Foxa1 (9-fold) in male mouse liver (orange bars). CAST is the regulating strain for this gene only in male DO mice (LOD = 10.41 in DO males with regression coefficient of -1.0). Significant male-biased expression is observed only in B6 (11-fold M/F) and not in CAST mouse liver. B. The female-biased gene Enpp1 is activated in B6 female liver, resulting in a gain of sex-biased expression in B6 mice (category #8 eQTL). This repression is associated with 4 potential genetic variants falling within a female-biased peak that is only female-biased in B6 (this is the only such region in the eQTL; see Sheet A in S6 Table). B6 is the regulating strain for this gene only in females and no strain is significant in male DO samples (LOD = 10.44 in CAST female with regression coefficient of -0.91) Significant female-biased expression is observed only in B6 (2.1-fold F/M) and not in CAST liver. C. The male-biased gene Bok is repressed in CAST female liver, resulting in a small increase in the magnitude of sex-bias in CAST liver (category #7 eQTL). This repression is associated with multiple genetic variants within a Bok intronic H3K27ac enhancer peak that is male-biased in CAST but is not sex-biased in B6 liver. CAST is the regulating strain for this gene only in female DO mice (LOD = 10.44 in DO females; regression coefficient, -0.91). The significant male-biased expression seen in B6 liver (2-fold M/F) is retained, and is moderately increased in CAST liver (2.3-fold F/M). Browser screenshot shows [file pgen.1009588.s010.pdf]

# S10AB Fig

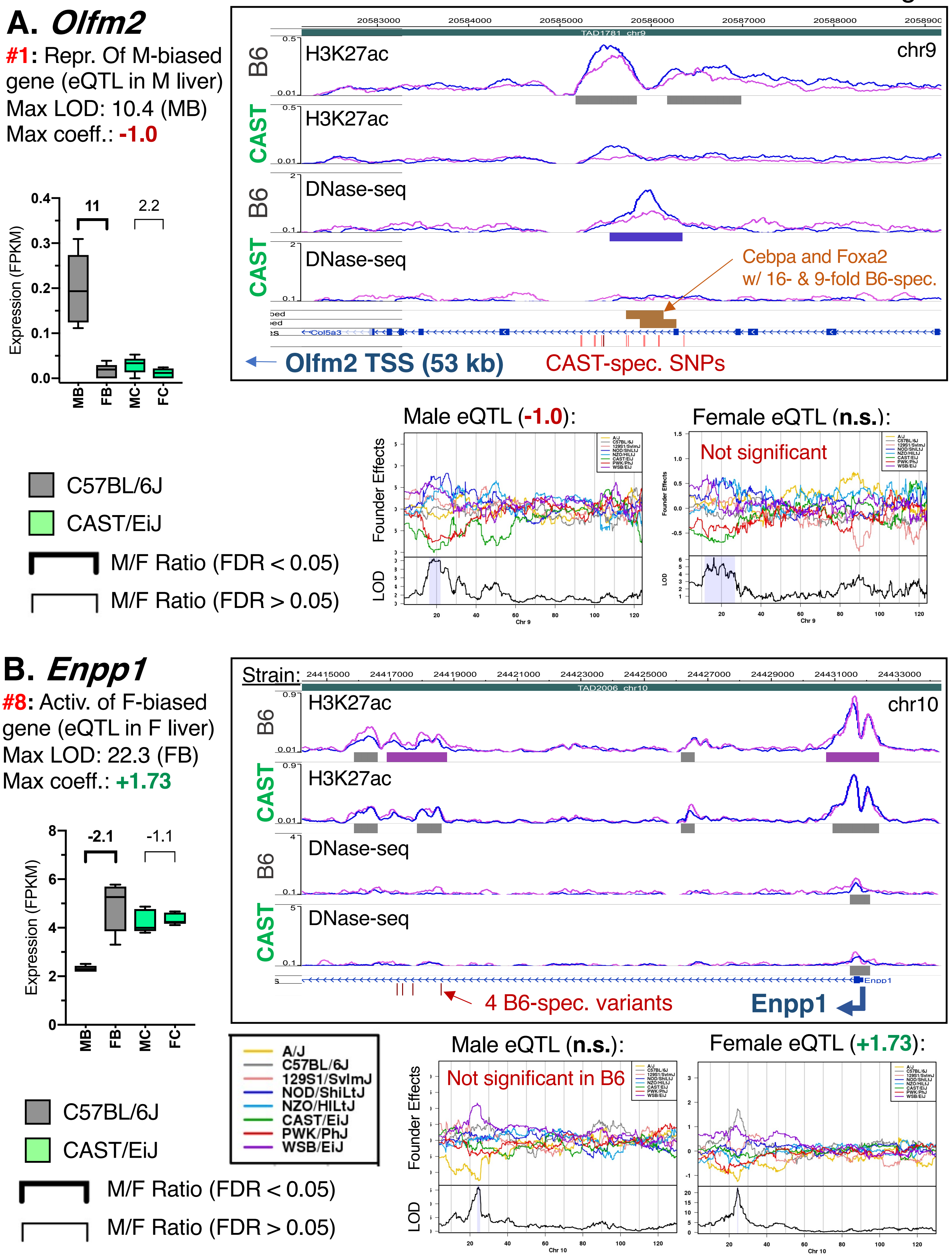

A. Olfm2
#1: Repr. Of M-biased gene (eQTL in M liver)
Max LOD: 10.4 (MB)
Max coeff.: -1.0
Expression (FPKM)
11
2.2
MB
FB
MC
FC
C57BL/6J
CAST/EiJ
M/F Ratio (FDR < 0.05)
M/F Ratio (FDR > 0.05)
20583000
20584000
20585000
20586000
20587000
20588000
TAD1781_chr9
chr9
B6
CAST
H3K27ac
DNase-seq
Cebpa and Foxa2
w/ 16- & 9-fold B6-spec.
Col5a3
Olfm2 TSS (53 kb)
CAST-spec. SNPs
Male eQTL (-1.0):
Female eQTL (n.s.):
Not significant
Founder Effects
LOD
Chr 9
B. Enpp1
#8: Activ. of F-biased gene (eQTL in F liver)
Max LOD: 22.3 (FB)
Max coeff.: +1.73
-2.1
-1.1
Strain:
24415000
24417000
24419000
24421000
24423000
24425000
24427000
24429000
24431000
24433000
TAD2006_chr10
chr10
Enpp1
4 B6-spec. variants
A/J
C57BL/6J
129S1/SvImJ
NOD/ShiLtJ
NZO/HILtJ
CAST/EiJ
PWK/PhJ
WSB/EiJ
Male eQTL (n.s.):
Female eQTL (+1.73):
Not significant in B6
Chr 10

S10CD Fig

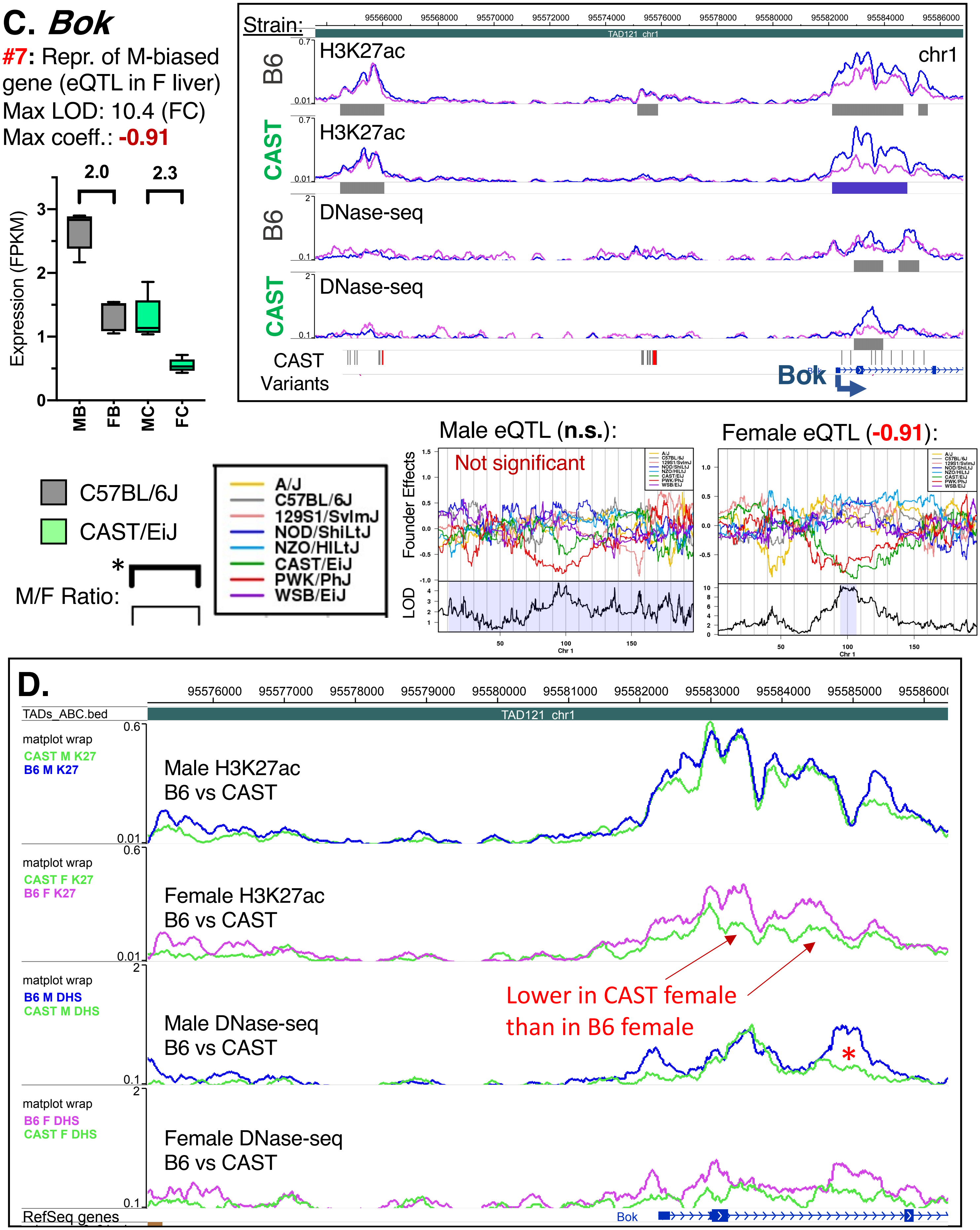

C. Bok
#7: Repr. of M-biased gene (eQTL in F liver)
Max LOD: 10.4 (FC)
Max coeff.: -0.91
2.0
2.3
Expression (FPKM)
MB
FB
MC
FC
C57BL/6J
CAST/EiJ
M/F Ratio:
*
A/J
C57BL/6J
129S1/SvImJ
NOD/ShiLtJ
NZO/HlLtJ
CAST/EiJ
PWK/PhJ
WSB/EiJ
Strain:
B6
CAST
H3K27ac
DNase-seq
chr1
TAD121_chr1
CAST Variants
Bok
Male eQTL (n.s.):
Female eQTL (-0.91):
Not significant
Founder Effects
LOD
Chr 1
D.
TADs_ABC.bed
matplot wrap
CAST M K27
B6 M K27
Male H3K27ac B6 vs CAST
CAST F K27
B6 F K27
Female H3K27ac B6 vs CAST
B6 M DHS
CAST M DHS
Male DNase-seq B6 vs CAST
B6 F DHS
CAST F DHS
Female DNase-seq B6 vs CAST
Lower in CAST female than in B6 female
RefSeq genes
