# Supplementary material for: Harnessing natural variation to identify cis regulators of sex-biased gene expression in a multi-strain mouse liver model
Source: PLoS Genet. 2021 Nov 9;17(11):e1009588. doi: 10.1371/journal.pgen.1009588 (PMC8664386; doi:10.1371/journal.pgen.1009588)

Rassf3, lnc9349, lnc9351 Activ. of M-biased genes in **CAST** F liver (cat.**#3**)

S12AB Fig

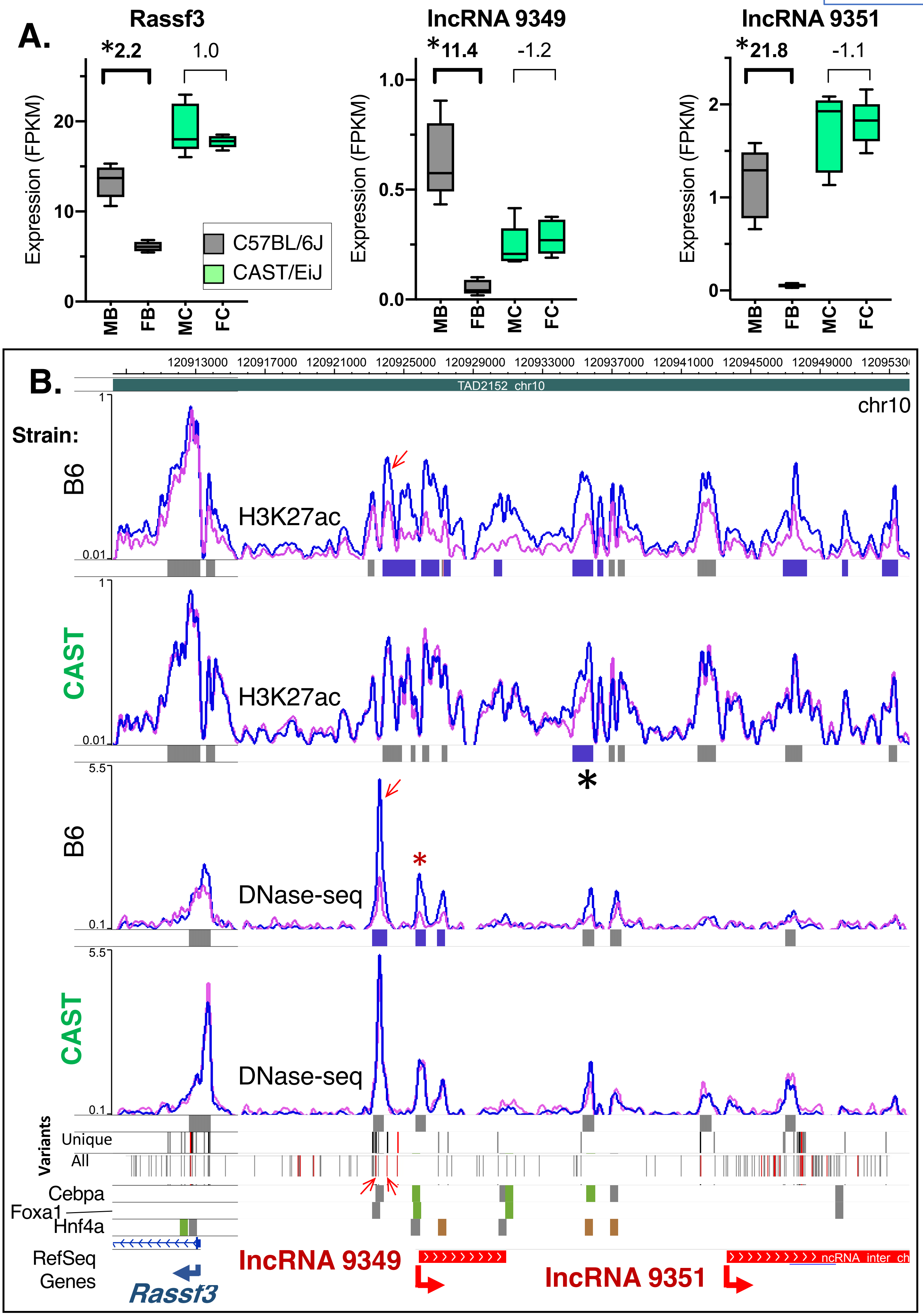

S12C-E Fig

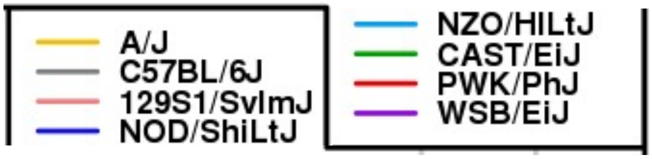

C. Rassf3

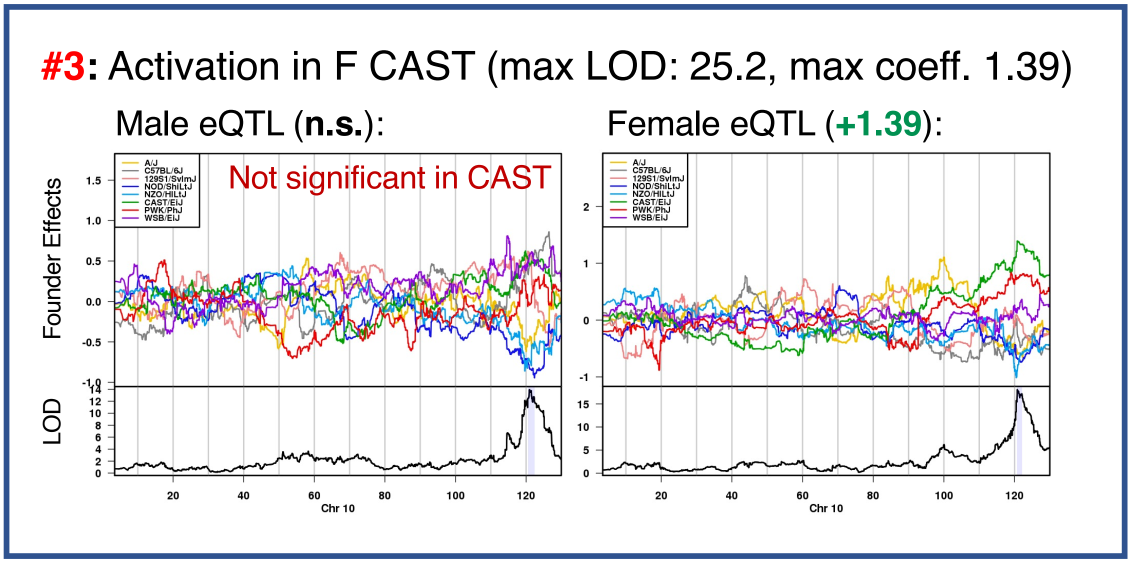

D. Lnc9349

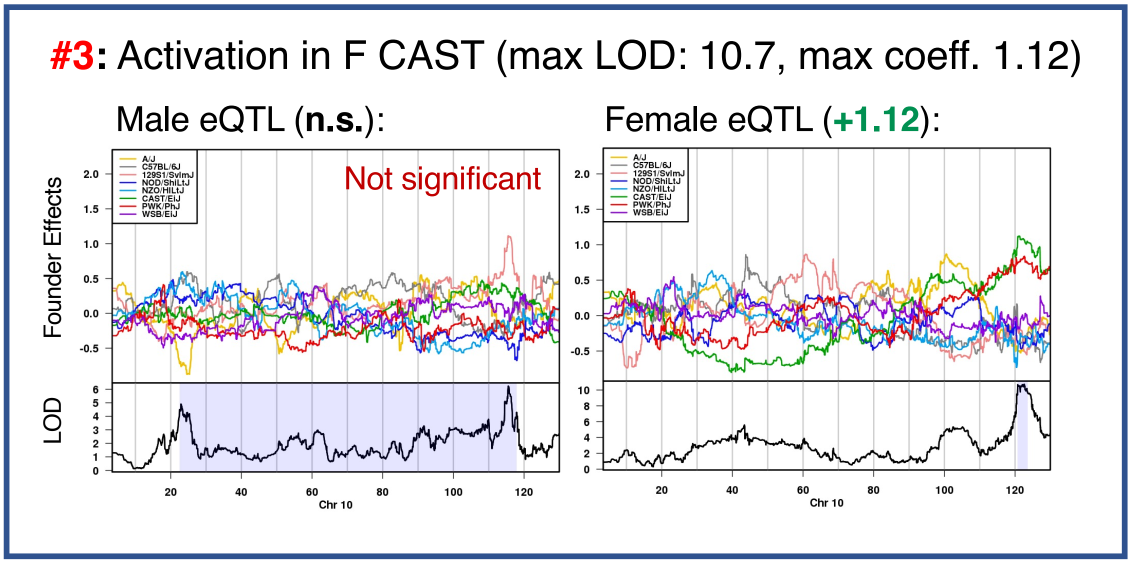

E. Lnc9351

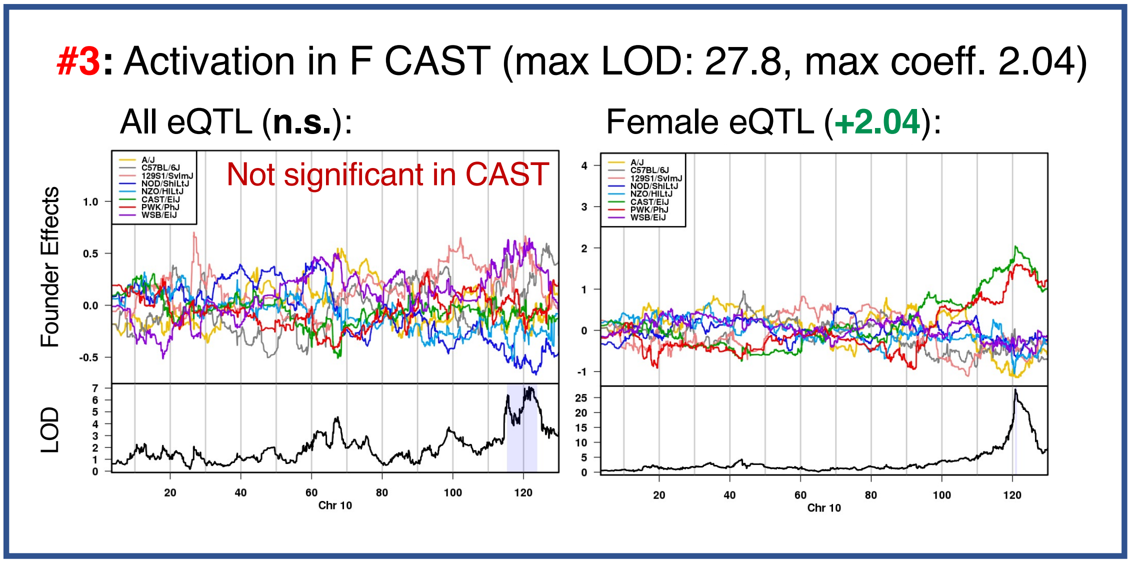

Supplement: S12 Fig — A. Three genes showing significant male-biased expression in B6 but not CAST liver. All three genes are up regulated by category #3 eQTLs, with CAST being the regulating strain in female but not male DO liver (S12 Fig), resulting in the loss of sex biased expression in CAST liver. *, significant M/F expression at FDR < 0.05. Data is presented as described in Fig 5. B. Browser screenshot showing multiple CREs male-biased in B6 but not CAST liver; only one CRE retains partial male bias in CAST liver (black asterisk). CAST Variants tracks: gray bars, single variants; red bars, multiple variants. Unique: strain-specific variants; All, all variants between B6 and CAST liver. Three other tracks indicate strain-specificity for transcription factor binding: green, CAST-preferential; orange, B6-preferential, as in Fig 7B. Red asterisk: DHS showing the relevant pattern of sex bias (loss of male bias in CAST) that lacks a strain-specific SNP/Indel. Red arrows, two CREs (K27ac and DHS) that show strain-specific sex dependence While the DHS at the TSS of lnc9349 does not contain a CAST-specific SNP/Indel, it does contain two SNPs that are shared between CAST and PWK, which are different from the mm9 reference allele (B6). Data is presented as described in Fig 5. C, D, E. eQTL analysis for three B6 mouse liver male-biased genes that loose sex-specific expression in CAST mouse liver. Rassf3, lncRNA9349 and lncRNA9351 all show male-biased expression in B6 mouse liver that is lost in CAST mice due to an activating eQTL seen in female, but not male DO mice (category #3: activation of a male-biased gene in female liver; Fig 4C), for which CAST was identified as the regulating strain, as seen here. No regulating strains were identified for male DO mice for lnc9349, and neither B6 nor CAST were the regulating strains for Rassf3 or lnc9351. Annotations and formatting are as described for Fig 5. (PDF) [file pgen.1009588.s012.pdf]
