# Supplementary material for: Harnessing natural variation to identify cis regulators of sex-biased gene expression in a multi-strain mouse liver model
Source: PLoS Genet. 2021 Nov 9;17(11):e1009588. doi: 10.1371/journal.pgen.1009588 (PMC8664386; doi:10.1371/journal.pgen.1009588)
Supplement: S13 Fig — Annotations and formatting are as described for Fig 5. A-C. eQTL analysis results for three sex-biased genes from chr10, with data presented as in Fig 5. Extensive strain effects are seen in both male and female for Gm4794 and Sult3a2 (positive coefficients in male and female DO mice with CAST as regulating strain, but stronger in male liver), whereas Rsph4a shows significant negative regulation in female but not male liver with CAST as regulating strain. D. Zoomed in screenshot for the Sult3a1 region (gene body and ~10 kb upstream) shown in Fig 8B, to better visualize the female-biased enhancer activity unique to CAST mice. Red asterisks indicate CREs showing strain bias but that lack any strain-specific SNPs/Indels (see Discussion). E. Zoomed in screenshot for the Rsph4a promoter region shown in Fig 8B, to better visualize the female-biased promoter activity unique to B6 mice. Of several CAST-specific variants within the B6-specific female-biased H3K27ac peak, one variant disrupts an HNF6 binding motif. This is also the only HNF6 motif within a female-biased HNF6 binding site in CD-1 liver in this eQTL region. We hypothesize that disruption of this motif results in observed loss of female-biased expression in CAST mouse liver seen in Fig 8A. (PDF) [file pgen.1009588.s013.pdf]

S13A-S13C Fig

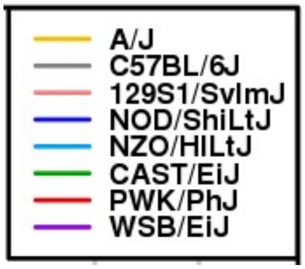

A. Gm4795 (Sult3a2)

#2: Activation in All CAST (max LOD: 42.1, max coeff. 2.50)

Male eQTL (**+2.50**):

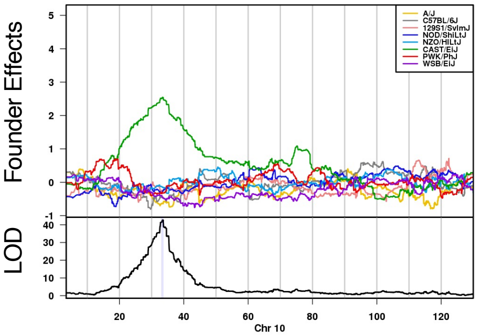

Female eQTL (**+1.05**):

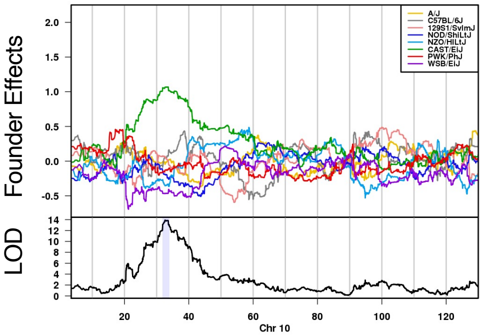

B. Sult3a1

#2: Activation in All CAST (max LOD: 41.1, max coeff. 2.47)

Male eQTL (**+2.47**):

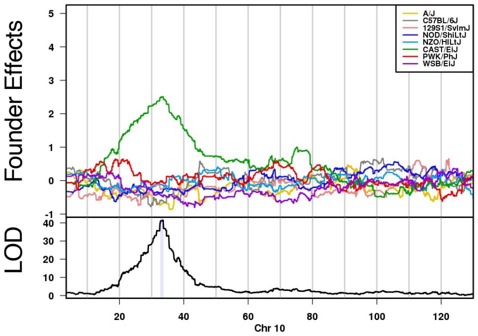

Female eQTL (**+0.85**):

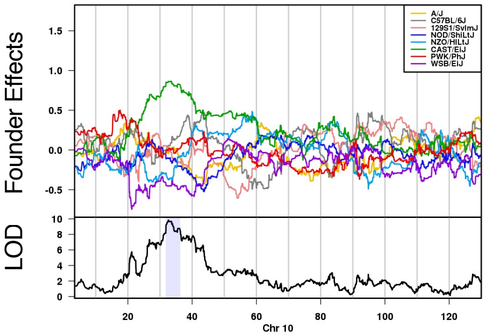

C. Rsph4a

#4: Repression in F CAST (max LOD: 15.1, max coeff. -1.01)

Male eQTL (**n.s.**):

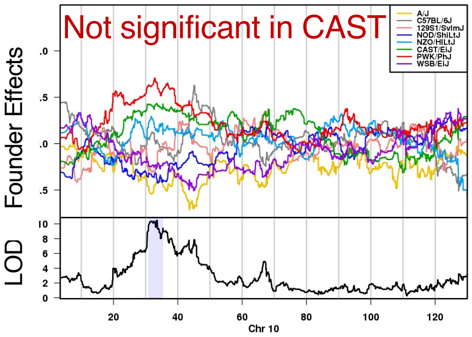

Female eQTL (**-1.01**):

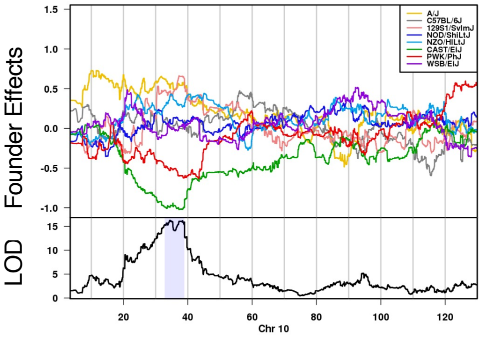

S13D Fig

D.

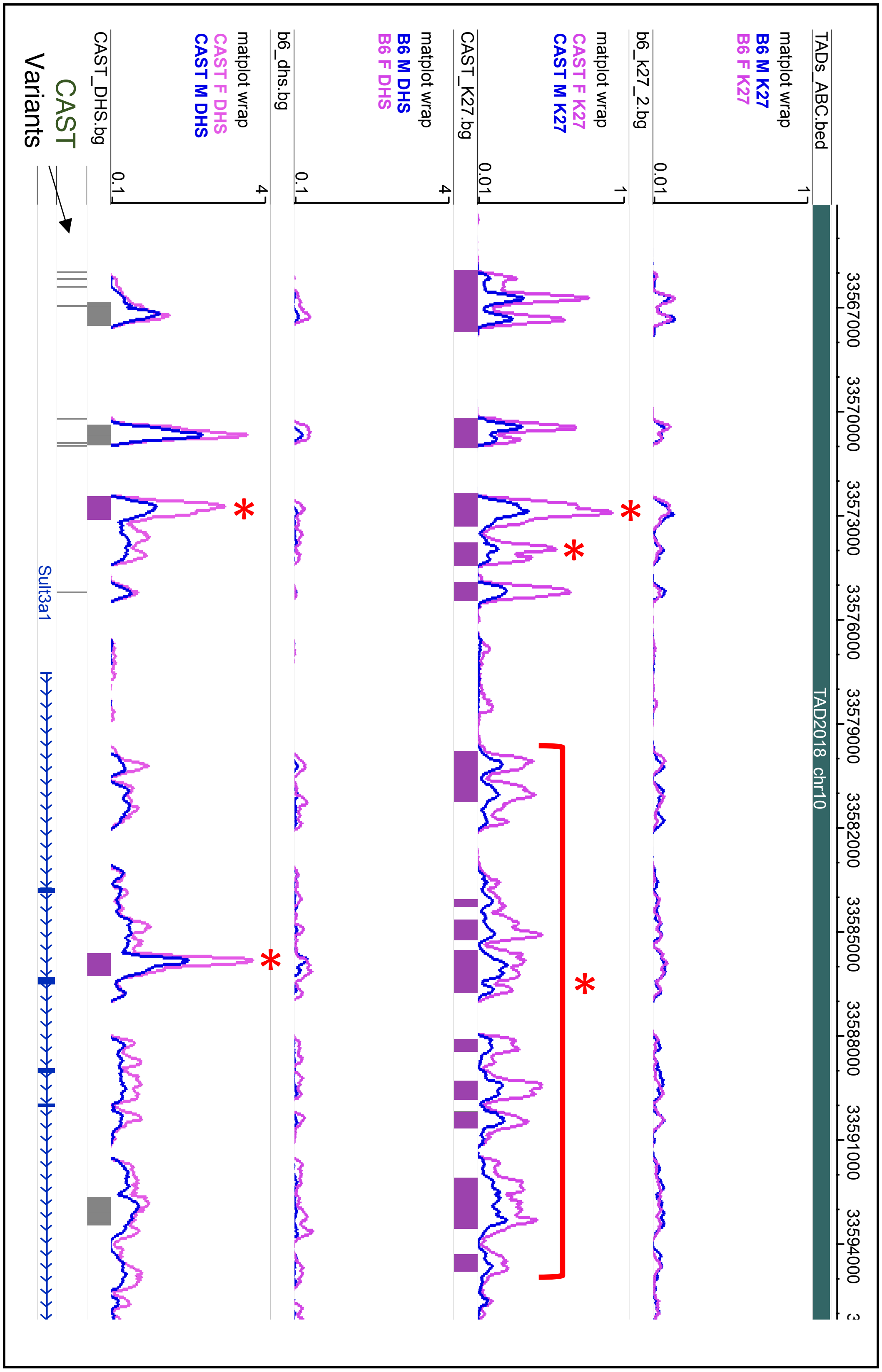
TADs_ABC.bed
TAD2018_chr10
matplot wrap
B6 M K27
B6 F K27
b6_k27_2.bg
matplot wrap
CAST F K27
CAST M K27
CAST_K27.bg
matplot wrap
B6 M DHS
B6 F DHS
b6_dhs.bg
matplot wrap
CAST F DHS
CAST M DHS
CAST_DHS.bg
CAST
Variants
Sult3a1
33567000
33570000
33573000
33576000
33579000
33582000
33585000
33588000
33591000
33594000

E.

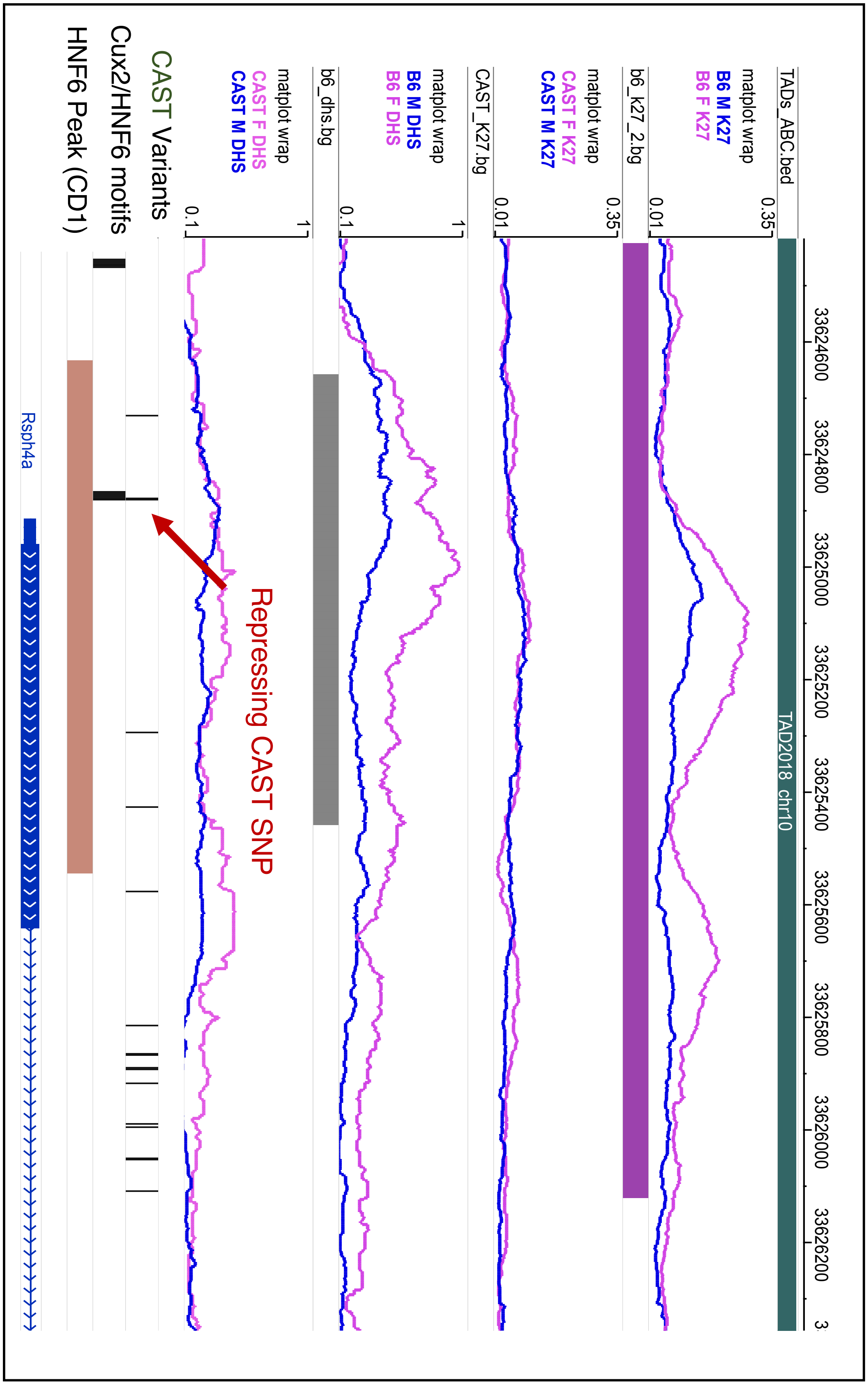
TADs_ABC.bed
TAD2018_chr10
matplot wrap
B6 M K27
B6 F K27
b6_k27_2.bg
matplot wrap
CAST F K27
CAST M K27
CAST_K27.bg
matplot wrap
B6 M DHS
B6 F DHS
b6_dhs.bg
matplot wrap
CAST F DHS
CAST M DHS
CAST Variants
Cux2/HNF6 motifs
HNF6 Peak (CD1)
Rsph4a
Repressing CAST SNP
33624600
33624800
33625000
33625200
33625400
33625600
33625800
33626000
33626200
