# Supplementary material for: Harnessing natural variation to identify cis regulators of sex-biased gene expression in a multi-strain mouse liver model
Source: PLoS Genet. 2021 Nov 9;17(11):e1009588. doi: 10.1371/journal.pgen.1009588 (PMC8664386; doi:10.1371/journal.pgen.1009588)
Supplement: Attachment — Submitted filename: Author responses_Final.docx [file pgen.1009588.s020.docx]

**Author’s response letter (PLoS Genetics #PGENETICS-D-21-00615)**

**Please note here if the review is uploaded as an attachment.**

**Reviewer #1**: In their manuscript “Harnessing natural variation to identify cis regulators of sex-biased gene expression in a multi-strain mouse liver model”, Matthews and Waxman identify potentially causal relationships between eQTLs, sex-biased CREs, and sex-biased gene expression in B6 and CAST mice. This is an extension of their previous work identifying eQTLs for sex-biased protein coding genes and lncRNAs. Here, they identify a core group of strain-conserved sex-biased genes that are proximally regulated, have greater sex bias, and are related to liver function. In comparison to conservation of sex-biased gene expression, the fraction of sex-biased CREs conserved across strains is much lower and there is little sequence conservation between those that are strain-shared, indicating that the mechanism of gene expression regulation is not explained by sequence alone and may differ by strain. This work provides insight into epigenetic regulation of sex-biased gene that can be easily extended in translational work to uncover sex-specific responses to liver disease and treatments in humans.

**Major Concerns:**
*1. There is one concern regarding the statistical methods used for the eQTL analysis. The identification of eQTLs in female and male mice is done by separate analysis of the samples by sex. Doing so reduces power and increases the chances that there observed sex differences are due to type II error. Instead, a sex-QTL interaction term should be included in a model using the full data.*

Author response: Thank you for the very helpful comment. We have now rerun the eQTL analysis to include a sex-QTL interaction term and address the concern that we may be overestimating the number of eQTLs with a significant sex difference (see Methods, **lines 865-880**). This reanalysis identified 168 eQTLs that meet this more stringent definition of sex-biased eQTLs. Importantly, all of the specific examples highlighted in our Figures were found to be significant by this reanalysis.

The main text was updated in the following specific ways:

-The text was updated to reflect this reanalysis and its effect on the number of eQTLs and characterize the genes that were removed by the reanalysis (**lines 290-299**).

-The eQTLs highlighted in Figures 5-8 were significant in both analyses, which is now noted in the text (**line 349**)

- We now designate the full set of eQTLs “lenient eQTLs”, to contrast with the “robust eQTLs’ that were identified as significant in both analyses.

-Supplemental Fig. S9B was added to characterize the effect of a more stringent/robust analysis on the degree of sex bias and expression of eQTL target genes.

-Supplemental Table S6 was updated with additional columns to indicate the LOD score for each model and whether the eQTL x sex interaction was significant (α of 0.05; ΔLOD of 4.27).

-The Methods section was updated with a new subsection “Reanalysis of sex-biased eQTLs by explicitly modeling sex-QTL interaction” (**lines 865-880**).

- The manuscript authorship was revised to add Dr. Tisha Melia as a co-author, given her contribution in performing these additional analyses, needed to address the Reviewer’s concern.

*2. Another shortcoming of the analysis is the filtering of genetic variants to those that are strain-specific. This limitation was addressed by the authors in the discussion (line 552-554), but relaxing this constraint to include any variants that differ between B6 and CAST would provide a more complete picture of potential causal regulators and help clarify cases where no strain-specific variant was identified within the CRE*.

Author response: The present study was designed as a follow-up to our prior published work (Melia and Waxman, PLoS One 2020) where eQTLs related to sex bias were identified for each of 8 DO mouse founder strains in a strain-dependent manner. As those eQTLs formed the basis for the current study, we faced the choice of either generating epigenomic datasets for males and females from all 8 DO mouse founder strains (which was not feasible due to limitations of time and funding), or alternatively, we could focus this follow up study on the two most distally-related DO founder strains (B6 and CAST). For practical reasons, we chose the latter approach, and given that the prior study had defined strain-specific sex-biased eQTLs, we limited the variants considered here to strain-specific variants while acknowledging clearly (as the Reviewer noted) that our findings represent a fraction of all possible segregating variants between B6 and CAST mice.

Two key points: First, our decision to focus on genetic variants that are unique to either B6 or CAST as compared to all 8 DO founder strains (strain-specific genetic variants) has the beneficial effect of greatly limiting the number of SNPs to be considered within an eQTL region, as can now be seen in the revised Variants tracks of Figs. 5-8 (Unique vs All variants tracks). Second, and more importantly, we believe that the approach we have taken is appropriate for a majority (70-74%) of eQTLs examined here, for which there is only one major strain that contributes to the eQTL (125 of the 168 robust sex-biased eQTLs; and 344 of the full set of 491 eQTLs; Table S6A). Thus, for these eQTLs, SNPs between B6 and CAST mice that are not strain-specific across the 8 DO mouse strains are less likely to be responsible for the strain differences in expression between B6 and CAST mice, given the strain-specific nature of the eQTL.

Thus, the limitation that we discussed in the paragraph that began on line 551-552 and was noted by the Reviewer, primarily applies to the 26-30% of eQTLs where two or more of the 8 strains contribute the eQTL, where we do need to consider the full set of SNPs between B6 and CAST mice. We have now done that and have included the full set of SNPs in the revised versions of Figs. 5-8, where all variants that segregate CAST and B6 are displayed (tracks marked All variants).

We have also made related modifications to the Results **(lines 359-360, 419-423**) and to the Discussion **(lines 580-588**) to discuss this issue explicitly and take it into account in our analysis and interpretation. Finally, we have softened our conclusions in several places regarding the inferences that can be drawn from our findings, including from CREs with no apparent SNPs in them (**lines 56, 340, 346, 462, 522**).

*3. The examples illustrating the gain or loss of a single sex-biased CRE provide compelling biological evidence of a causal relationship between the eQTL, CRE, and gene expression. For these examples, regressing gene expression on the eQTL while conditioning on the nearby H3K27ac peak and/or DHS could provide further evidence that the mechanism by which the eQTL is influencing gene expression is epigenetic modifications at the CRE. Doing the same for examples where there are multiple sex-biased CREs may also elucidate which CREs are regulators of sex-biased gene expression, and which may be reacting independently to the QTL.*

Author response: Unfortunately, we do not have a suitable experimental dataset to perform the additional analysis that the Reviewer suggests, as we believe the current study is underpowered for such analysis. Specifically, the Reviewer suggests that to further support our conclusion that epigenetic changes at CREs regulate expression, we might test whether the presence, and the intensity, of epigenetic marks at the eQTL region correlate with gene expression. To implement that, we would need to build a model, where gene expression is predicted by eQTL (genotypes) and nearby epigenetic features (chromatin marks/DHS). If the nearby chromatin marks or DHS correlate with gene expression, the significance of the eQTL will disappear (meaning, chromatin marks or DHS are as good as genotypes when predicting the gene expression level). Modeling it this way can potentially help determine which CRE is the best predictor of the observed gene expression. However, in practice, to do this, we would need to assay, for a sufficiently large number of individual livers and mouse genotypes, gene expression and hepatic chromatin marks/DHS for each individual liver. Ideally, these analyses would be performed using DO mice, as the range of genetic diversity is extremely limited if we only consider CAST and B6 mice. However, our epigenetic datasets are limited to 4 individual mice for each of 2 sexes and representing only 2 genotypes, which is, unfortunately, is far too few samples to build a reliable model.

**Minor Concerns:**
*4. There is no color in the legend for figure B and C*

Author response: The Figure Legends for Fig. 1 have been updated to show the correct color scales.

*5. It is best practice to show differences in means with points and error bars instead of bar charts with error bars (Krzywinski and Altman, 2014). Thus, Figure S5A-C should show points with error bars instead of the bars*.

Author response: Figure S5A-C graphs have been replaced with plots indicating individual values in addition to the median and 1.5*IQR values.

*6. Replace C57Bl/6J with C57BL/6J throughout*.

Author response: These typos have been corrected throughout the text.

**Reviewer #2**: This is an interesting paper that carefully analyzes a rich data set to better understand the interactions between sex, genetic variants, and gene regulation in the liver. I’ve included a number of suggestions and a few concerns. Chief among them is point #19 – the absence of some SNPs that segregate between the two strains under study fundamentally changes the results. The second concern is implied causality, which could largely be addressed through softening some language or moving interpretation to the discussion section.

Intro
*1. Line 129 – some will quibble with the purity of the inbred strain subspecies assignment. The best estimates are in the 90+% range. See Yang 2011. The authors might simply write “mostly M.m.domesticus” instead.*

Author response: The text was altered to indicate that B6 is an inbred laboratory mouse strain “composed of primarily subspecies *Mus musculus domesticus* genetic background” (**lines 131, 157-158**).

Section 1
*3. What is the analysis for the statement “showing the same expression pattern” (line 158)? For example, what separates FA, FB2, and FC1? There are no methods described for cluster assignments.*

Author response: We have updated the Figure legend and the methods to specify more clearly the method used for clustering (hierarchical clustering by Euclidean distance) **(lines 987-988, 1033**).

*4. The heatmap suggests there is higher variance among replicates for FB2 – is there a way to quantify that?*

Author response: We calculated the per-gene coefficient of variance (CV) among replicates for group FB2 and FA for comparison. The median CV for FB2 was indeed higher than FA (median CV of 32.9 versus 31.9, respectively), however this difference was not significant (p= 0.0991; student’s T). The difference was greater comparing the means of each group: mean CV for FA=36.2 while mean CV for FB2=43.1. To us this suggests that for individual genes there may be a difference in variation, but as a group there is not significantly higher variation in expression of genes in cluster FB2 than those in cluster FA.

*5. Cd-1 is included inconsistently – present in Fig 1A, absent in Fig 1B/C. The text mirrors this – Cd-1 mentioned in only one sentence but since it is the second sentence of the result it sets expectations for some treatment of those results. For instance, is there higher sharing between B6 and CD-1 because of subspecies similarity?*

Author response: Yes, the subspecies similarity is the main driver of the greater degree of sharing between CD-1 and B6 compared to CAST. CD-1 is primarily included as sex differences have been most extensively characterized in this strain. We have highlighted this information more prominently in this section on **lines 110-112** and **lines 155-158**.

*6. “Strain-shared” was confusing the first time I read it (partly because there are three strains). In Results Section 3 you switch to “strain-conserved.” Define the terms clearly up font.*

Author response: Thank you for highlighting this, we have provided a clearer definition the first time this phrase is introduced on **line 160**: “defined as significant sex bias in both B6 and CAST”

*7. Figure captions are very long and have info that could go in methods or results.*

Author response: We have edited the figure legends wherever possible, to eliminate details that are not essential or are redundant with the Methods or Results text, or in some cases could be moved to the Methods, as the Reviewer suggested. We view it important that Figure legends provide sufficient information for the reader to know what is actually displayed, for maximum transparency.

*8. The references to the figures in the text help immeasurably (e.g. “MA” and “FA”).*

Author response: When possible, the text was modified to explicitly point to clusters in Fig 1B/C to highlight points being made in the Results section. For example, **lines 167-169** were rewritten as follows:

The remaining 71% of strain-shared sex-biased genes showed strain differences in the absolute expression level (FPKM) and magnitude of sex bias of the other core sex-biased genes (e.g., genes in clusters MB, FB1, and FB2 showed higher expression in B6 than CAST).

Annotation was also added to **lines 260**.

Section 2
9. Main point on line 179 is interesting and potentially important. Think about leading the section with that result, since line 169-172 supports that statement. (minor)

Author response: The conclusion statement has been moved to the first line of the section “Functional roles of strain-shared vs strain-unique sex-biased genes”, with some minor reformatting (**lines 177-179**).

Section 3
*10. Analysis for statement in line 219?*

Author response: This is based on the analysis shown in Fig. S6G, as now noted (line 219).

Section 4
*11. Is line 249 the first appearance of the 43 strain-shared sex-based genes with consistent expression? This seems to be relevant right out of the gate (i.e. results section 1). Also related to the clustering question maybe?*

Author response: Yes, this is another case relevant to point #8 where referencing cluster names is helpful. We have added cluster annotations to clarify this point (Lines 260-261).

Section 5
*12. The categories (line 308) are not described in the text. If this is all coming from a previous publication with Diversity Outbred data, it would be nice to describe those results in brief. Fig4C is useful but the reader is forced to stop reading in the middle of a paragraph in order to find that figure and make sense of the results.*

Author response: The text on **lines 329** and **lines 332-334** has been revised to highlight our prior study where these categories were defined, and to outline the category definitions in brief, while pointing the reader to the Figure for specific details. Additional text underlined, below.

*13. I also wonder if there isn’t a better way to categorize than LOD scores from eQTL mapping? These are test statistics and dependent on a number of factors, especially the allele frequencies (maybe diplotype frequencies in the DO) and within group variance (measurement error but also genetic background). In any case, if we map an eQTL in male result with confidence of p ≤ 0.1 and map the eQTL in females with confidence of p≤0.0001, I don’t believe that means the eQTL is “stronger” in Females – just that I was lucky enough to map it with more confidence. It also seems that you can compare gene expression levels directly between DO males and females, accounting for eQTL genotype, and avoid this problem.*

Author response: This comment is similar to the first point made by Reviewer 1, which led us to reanalyze the eQTLs identified in a previous study (Melia and Waxman 2020) with a new model explicitly considering sex as a covariate and identify a more robust set of eQTLs and increase the confidence for this set. The new results are integrated into the revised manuscript (**lines 290-299** and elsewhere)

*14. What are “the figures below”? Line 326*

Author response: The text has been updated to reference the specific Figures in question (**lines 349-350**):

*15. Is the contention that the local eQTL are in fact correlated with sex-biased CREs even though the casual SNP is not actually in the CRE (and we know this because there is little variation per the previous set of results)?*

Author response: There is variation within CREs at approximately the rate expected if they were randomly distributed throughout the genome, thus there are many variants located within CREs. However, while this provides correlative evidence that specific CREs may regulate a given gene, it is difficult to determine the precise mechanism of action as they are generally not motif-disrupting (i.e., they do not implicate specific transcription factors involved).

*16. I am not sure I understand the causality here – you propose that the variant + sex controls the CRE which in turn controls gene expression, but there are alternative models that are confounded. This section is a little hard to follow – at the very least, the authors should make sure they do not confuse correlation with causation here. Generally, The results in 323-357 are interspersed with interpretation that may be more appropriate for the discussion section. Certainly, the data are consistent with some of the mechanistic models the authors describe but the data don’t prove causation.*

Author response: We have softened some of the language so as not to overstate the correlative nature of this analysis. This includes statements such as “although correlative in nature” (**line 346**) and “can potentially be explained” (**line 340**). We have also attempted to highlight the additional covariates considered in our robust eQTL model (specifically sex, DO generation, genotyping batch, diet and sex*diet) which encompasses many, but certainly not all, potential confounding variables.

Section 6-8
*17. The last four pages of results, along with 5 large figures, consist of specific examples of genes, eQTLs, and their regulation patterns. These are probably useful but distract from the “big picture” results that have been described up to that point. This is likely a matter of preference, but I very much enjoyed the first 4-5 sections of the results and found the last three a slog. The authors may consider condensing these results or moving some parts to supplemental material.*

Author response: We do see the Reviewer’s point and have now moved one of the figures to the Supplemental material (prior Fig. 8, now integrated into Fig. S12). We feel it important to include the remaining figures to make the general points exemplified by these genomic regions, and to give the reader an opportunity to appreciate the underlying raw datasets supporting this study and its major conclusions.

Discussion/Methods
*18. The discussion places each result in the context of gene annotations and related literature.*

Author response: (no specific response required)

*19. The paragraph starting in line 551 is potentially important. Why would the analysis not include all SNPs that segregate between CAST and B6? This undercuts the statements made in the results that there are CREs with no SNPs in them. This is a major concern with the analysis given the importance that it has on the results.*

Author response: The present study was designed as a follow-up to our prior published work (Melia and Waxman, PLoS One 2020) where eQTLs related to sex bias were identified for each of 8 DO mouse founder strains in a strain-dependent manner. As those eQTLs formed the basis for the current study, we faced the choice of either generating epigenomic datasets for males and females from all 8 DO mouse founder strains (which was not feasible due to limitations of time and funding), or alternatively, we could focus this follow up study on the two most distally-related DO founder strains (B6 and CAST). For practical reasons, we chose the latter approach, and given that the prior study had defined strain-specific sex-biased eQTLs, we limited the variants considered here to strain-specific variants while acknowledging clearly (as the Reviewer noted) that our findings represent a fraction of all possible segregating variants between B6 and CAST mice.

Two key points: First, our decision to focus on genetic variants that are unique to either B6 or CAST as compared to all 8 DO founder strains (strain-specific genetic variants) has the beneficial effect of greatly limiting the number of SNPs to be considered within an eQTL region, as can now be seen in the revised Variants tracks of Figs. 5-8 (Unique vs All variants tracks). Second, and more importantly, we believe that the approach we have taken is appropriate for a majority (70-74%) of eQTLs examined here, for which there is only one major strain that contributes to the eQTL (125 of the 168 robust sex-biased eQTLs; and 344 of the full set of 491 eQTLs; Table S6A). Thus, for these eQTLs, SNPs between B6 and CAST mice that are not strain-specific across the 8 DO mouse strains are less likely to be responsible for the strain differences in expression between B6 and CAST mice, given the strain-specific nature of the eQTL.

Thus, the limitation that we discussed in the paragraph that began on line 551-552 and was noted by the Reviewer, primarily applies to the 26-30% of eQTLs where two or more of the 8 strains contribute the eQTL, where we do need to consider the full set of SNPs between B6 and CAST mice. We have now done so, and have included the full set of SNPs in the revised versions of Figs. 5-8, where all variants that segregate CAST and B6 are displayed (tracks marked All variants).

We have also made related modifications to the Results **(lines 359-360, 419-423**) and to the Discussion **(lines 580-588**) to discuss this issue explicitly and take it into account in our analysis and interpretation. Finally, we have softened our conclusions in several places regarding the inferences that can be drawn from our findings, including from CREs with no apparent SNPs in them (**lines 56, 340, 346, 462, 522**).

**Have all data underlying the figures and results presented in the manuscript been provided?**
Large-scale datasets should be made available via a public repository as described in the *PLOS Genetics* [data availability policy](http://journals.plos.org/plosgenetics/s/data-availability), and numerical data that underlies graphs or summary statistics should be provided in spreadsheet form as supporting information.

Reviewer #1: Yes

Reviewer #2: Yes

PLOS authors have the option to publish the peer review history of their article ([what does this mean?](https://journals.plos.org/plosgenetics/s/editorial-and-peer-review-process#loc-peer-review-history)). If published, this will include your full peer review and any attached files.


If you choose “no”, your identity will remain anonymous but your review may still be made public.


**Do you want your identity to be public for this peer review?** For information about this choice, including consent withdrawal, please see our [Privacy Policy](https://www.plos.org/privacy-policy).

Reviewer #1: **Yes:** Gary A Churchill

Reviewer #2: No
