# Supplementary material for: Harnessing natural variation to identify cis regulators of sex-biased gene expression in a multi-strain mouse liver model
Source: PLoS Genet. 2021 Nov 9;17(11):e1009588. doi: 10.1371/journal.pgen.1009588 (PMC8664386; doi:10.1371/journal.pgen.1009588)
Supplement: Attachment — Submitted filename: Authors_response to Editors final request.docx [file pgen.1009588.s021.docx]

Oct 25, 2021

Dear Editor,

We have responded to your specific request by revising the manuscript text, as follows:

**Editor’s specific request**

*The manuscript was fully evaluated at the editorial level and by independent peer reviewers. The reviewers appreciated the attention to their concerns and overall were satisfied by the manner in which they were addressed. There was just one remaining point raised by the second reviewer that we believe needs attention. This issue pertains to the assignment of gains and losses within a specific strain. In most cases, it is likely accurate to assume that the ancestral allele matches the allele present in the majority of the founder strains. It is possible, though, that the ancestral allele matches the private allele in the single strain, and that the change occurred on a branch (or independently on multiple branches) that included the majority seven founder strains. This would be highly unlikely for B6, but it is not outside the realm of possibility for CAST.*

*We believe that this issue could be efficiently addressed in the text and would not need additional analyses, but we encourage the authors to do so. This is the only issue that needs to be addressed.*

We have responded by adding the following explanatory text to the Methods and to the Discussion, and we have cross referenced these points in the Results, as follows:

**Methods, lines 864-868**

**Definitions for regulating strain and gain or loss of sex bias.** The term “regulating strain” is used to indicate the strain in which an eQTL was identified with the most significant LOD score. The terms “gain” and “loss” of sex bias in the regulating strain are based on the assumption that any private genetic variant (i.e., one unique among all DO founder strains) differs from the ancestral allele.

**Methods, lines 944-951**

As noted above, the terms loss and gain of sex-specific gene expression pattern used here are predictions based on the observed change of sex-specific gene expression pattern in the regulating strain, as compared to other strains examined, and the specific category of eQTL we discovered (categories #1-8). However, absent knowledge of the true ancestral allele, we have no way to verify these assignments. Accordingly, a subset of the alleles that we have designated as a gain of sex-specific gene expression could in fact be a loss of sex-specific gene expression pattern in multiple DO founder strains, and vice versa.

**Discussion, lines 579-586**

The nomenclature of “gain” and “loss” of sex bias used throughout this study is based on the underlying assumption that the private/unique genetic variants that are the focus of this study are unlikely to be shared between the regulating strain and the ancestral state (see Methods for additional definition of these terms). While we consider this assumption most probable, it remains a possibility that the ancestral allele and even an allele that is strain-specific among DO founder strains are shared. This is more likely for CAST mice than for B6 mice, given its evolutionary distance from the other founder strains. Further analysis of several additional wild-derived mouse strains more closely related to CAST would be required to further evaluate this issue.

**Discussion, lines 597-602**

Further, as noted above, we cannot validate our assignments of loss and gain of sex-specific gene expression alleles without knowing the true ancestral allele. A loss of gene expression pattern in one strain could be a gain of gene expression pattern in multiple DO founder strains, a case more likely to occur when the mutation is acquired prior to a speciation event, for example, speciation of the six *M. m. domesticus* strains examined here.

**Results**

**Line 284**: regulating strain (see Methods for definition)

**Line 332**: gain or loss of sex specificity in the regulating strain (see Methods for definitions)

I trust that you will now find our manuscript acceptable for publication in PLoS Genetics.

Sincerely,

David J Waxman
